# Nanozymes with Peroxidase-like Activity for Ferroptosis-Driven Biocatalytic Nanotherapeutics of Glioblastoma Cancer: 2D and 3D Spheroids Models

**DOI:** 10.3390/pharmaceutics15061702

**Published:** 2023-06-10

**Authors:** Sandhra M. Carvalho, Alexandra A. P. Mansur, Izabela B. da Silveira, Thaisa F. S. Pires, Henrique F. V. Victória, Klaus Krambrock, M. Fátima Leite, Herman S. Mansur

**Affiliations:** 1 Center of Nanoscience, Nanotechnology, and Innovation—CeNano2I, Department of Metallurgical and Materials Engineering, Federal University of Minas Gerais, UFMG, Belo Horizonte 31270-901, Brazil; sandhra.carvalho@gmail.com (S.M.C.); alexandramansur.ufmg@gmail.com (A.A.P.M.); thaisafsantos@gmail.com (T.F.S.P.); 2 Department of Physiology and Biophysics, Institute of Biological Sciences—ICB, Federal University of Minas Gerais, UFMG, Belo Horizonte 31270-901, Brazilleitemd@icb.ufmg.br (M.F.L.); 3 Department of Physics, Federal University of Minas Gerais, UFMG, Belo Horizonte 31270-901, Brazil; henrivic@hotmail.com (H.F.V.V.); klauskrambrock@yahoo.com.br (K.K.)

**Keywords:** nanoparticles, tumor targeting, cancer, nanotherapeutics, nanotheranostics, nanohybrids, polymer–peptide bioconjugates, nanozyme biocatalytic

## Abstract

Glioblastoma (GBM) is the most common primary brain cancer in adults. Despite the remarkable advancements in recent years in the realm of cancer diagnosis and therapy, regrettably, GBM remains the most lethal form of brain cancer. In this view, the fascinating area of nanotechnology has emerged as an innovative strategy for developing novel nanomaterials for cancer nanomedicine, such as artificial enzymes, termed nanozymes, with intrinsic enzyme-like activities. Therefore, this study reports for the first time the design, synthesis, and extensive characterization of innovative colloidal nanostructures made of cobalt-doped iron oxide nanoparticles chemically stabilized by a carboxymethylcellulose capping ligand (i.e., Co-MION), creating a peroxidase-like (POD) nanozyme for biocatalytically killing GBM cancer cells. These nanoconjugates were produced using a strictly green aqueous process under mild conditions to create non-toxic bioengineered nanotherapeutics against GBM cells. The nanozyme (Co-MION) showed a magnetite inorganic crystalline core with a uniform spherical morphology (diameter, 2R = 6–7 nm) stabilized by the CMC biopolymer, producing a hydrodynamic diameter (H_D_) of 41–52 nm and a negatively charged surface (ZP~−50 mV). Thus, we created supramolecular water-dispersible colloidal nanostructures composed of an inorganic core (Cox-MION) and a surrounding biopolymer shell (CMC). The nanozymes confirmed the cytotoxicity evaluated by an MTT bioassay using a 2D culture in vitro of U87 brain cancer cells, which was concentration-dependent and boosted by increasing the cobalt-doping content in the nanosystems. Additionally, the results confirmed that the lethality of U87 brain cancer cells was predominantly caused by the production of toxic cell-damaging reactive oxygen species (ROS) through the in situ generation of hydroxyl radicals (·OH) by the peroxidase-like activity displayed by nanozymes. Thus, the nanozymes induced apoptosis (i.e., programmed cell death) and ferroptosis (i.e., lipid peroxidation) pathways by intracellular biocatalytic enzyme-like activity. More importantly, based on the 3D spheroids model, these nanozymes inhibited tumor growth and remarkably reduced the malignant tumor volume after the nanotherapeutic treatment (ΔV~40%). The kinetics of the anticancer activity of these novel nanotherapeutic agents decreased with the time of incubation of the GBM 3D models, indicating a similar trend commonly observed in tumor microenvironments (TMEs). Furthermore, the results demonstrated that the 2D in vitro model overestimated the relative efficiency of the anticancer agents (i.e., nanozymes and the DOX drug) compared to the 3D spheroid models. These findings are notable as they evidenced that the 3D spheroid model resembles more precisely the TME of “real” brain cancer tumors in patients than 2D cell cultures. Thus, based on our groundwork, 3D tumor spheroid models might be able to offer transitional systems between conventional 2D cell cultures and complex biological in vivo models for evaluating anticancer agents more precisely. These nanotherapeutics offer a wide avenue of opportunities to develop innovative nanomedicines for fighting against cancerous tumors and reducing the frequency of severe side effects in conventionally applied chemotherapy-based treatments.

## 1. Introduction

Although undeniable scientific and technological developments have occurred in recent decades, providing remarkable achievements, regrettably, cancer remains a major lethal disease burden, with millions of deaths and newly diagnosed cases worldwide every year. Brain tumors are a relatively broad class of neoplasms, within which, according to the World Health Organization, glioblastoma (GBM, grade IV glioma) is the most recurrent form of brain cancer affecting the central nervous system, which frequently indicates a very poor prognosis for patients. Unlike other solid tumor cells, GBM widely and aggressively invades neighboring brain tissues [1,2,3,4].

Unfortunately, most brain cancer therapies predominantly rely on more conventional tools based on surgical resection followed by radiotherapy, with the concurrent maintenance of drug-mediated chemotherapy. Such a strategy is mostly focused on treating cancer relying on killing cells without achieving a high specificity, which frequently leads to systemic toxicity and severe side effects to patients, with prognoses typically ranging from only a few weeks to several months, often not surpassing 18 months [1,2]. In fact, no substantial progress has been made in advancing new chemotherapies for glioblastoma since the approval of the drug temozolomide in 2005 [2,5].

Fortunately, more recently, a fascinating novel field of research known as nanomedicine, combining nanotechnology with biology and medicine, has emerged as an incomparable strategy capable of overcoming the challenges posed in cancer diagnosis and therapy, ultimately accomplishing a full remission of the disease [1,6,7,8]. In this view, a broad arsenal of ground-breaking nanomaterials is engineered to start therapies with several characteristics for fighting against cancer (i.e., nanotheranostics) at the earliest possible stage of diagnosis. Undoubtedly, the major goal remains to kill cancer, but it has become progressively more evident how vital it is also to improve the quality of life of patients during treatment by reducing often harmful and disturbing side effects. This challenge is overcome by adopting a pivotal cancer nanomedicine strategy [1,9] using nanostructures for smart drug delivery systems that can precisely and effectively reach diseased sites with cancer cells at the very beginning of the formation of the tumor, while reducing the potential toxicity to healthy cells and tissues [1,10,11,12,13,14,15]. Cancer nanotechnology has expanded by combining nanomaterials, such as inorganic nanoparticles, with macromolecules, polymers, and drugs, forming nanostructures. These advanced hybrid nanostructures (named nanohybrids) amalgamate the best of each component to produce innovative solutions with unique functionalities integrated into “all-in-one” nanosystems, which cannot be otherwise achieved by any component separately. Hence, a breadth of new nanomaterials has been intensively studied combined with polymers and drug nanocarriers for cancer diagnosis and therapy, including inorganic fluorescent semiconductor quantum dots (e.g., binary, ZnS, ZnSe, InP; ternary, Cu-In-S, Ag-In-S), metallic nanoparticles (e.g., Au, Pt, Ag), iron-oxide-based nanoparticles (e.g., Fe_2_O_3_, Fe_3_O_4_, MFe_2_O_4_), and others [1,2,6,7,16,17,18,19,20].

Although many smart nanosized materials have been developed for cancer diagnosis and treatment, some have received special attention because they resemble the biocatalytic activity of natural enzymes. These enzyme-mimicking inorganic nanomaterials, called nanozymes, play a crucial role in the field of cancer nanomedicine for diagnosis and therapy. When compared to natural enzymes, nanozymes have drawn much attention because they have higher thermal and chemical stabilities, biosafety, superior catalytic efficiency, a moderately low cost, and are easy to prepare. Regarding nano-oncology therapy applications, nanozyme-based structures can kill malignant cells in two ways. The first is by directly killing cells through increasing reactive oxygen species (ROS) relying on the oxidase and peroxidase activities of the nanozymes. The second is through the indirect killing effect associated with the catalase (CAT) or superoxide dismutase (SOD) activity of nanozymes by altering the common hypoxia of the tumor microenvironment (TME) [3,21,22,23,24].

Usually, nanozymes are categorized into three major classes regarding their nature from a materials science perspective: noble nanometals (e.g., Au, Ag, Pt, Pd), nanoparticles made of metal-oxide-based compounds (e.g., Fe_3_O_4_, MFe_2_O_4_, CeO_2_), and carbon-based nanozymes (e.g., carbon dots, carbon nanotubes, and graphene oxide) [3,24]. Since the study of nanozymes pioneered by Gao and collaborators (2007) [21], iron-oxide-based nanomaterials have been intensively investigated as nanozymes among several options of enzyme-mimicking nanomaterials. This surprising discovery established for the first time that ferrite-based nanoparticles present an intrinsic peroxidase-like (POD) activity, which resembles the typical catalytic behavior observed on the natural enzyme horseradish peroxidase (HRP). Consequently, research on iron-oxide-based nanozymes has shown remarkable growth in the last decade, including cancer nanomedicines for therapy and nanotheranostics [3,24].

Magnetic iron oxide nanoparticles (MIONs), such as magnetite/maghemite (Fe_3_O_4_/γ-Fe_2_O_3_), possess important unique features besides their important magnetic behavior, which include physicochemical stability, biocompatibility, and comparatively low toxicity, which could also favor cancer therapy applications [1,25,26]. Because of their intrinsic properties, MIONs have been developed to integrate multiple functions for cancer diagnosis (e.g., bioimaging as an MRI contrast agent) and therapy, encompassing controlled drug delivery nanocarriers and magnetic hyperthermia. Although initial reports [21] posited that most ferrite-based nanoparticles are chemically inert and biologically non-toxic, this is not necessarily true. On the contrary, several reports have shown that they behave as nanozymes with enzyme-like biocatalytic activity in oxidative processes which can cause cytotoxicity [1,6,27,28,29,30,31,32].

Although not fully understood yet, the cell death mechanism promoted by iron-oxide-based nanoparticles, which are higher in magnetite (Fe_3_O_4_) and lower in maghemite (γ-Fe_2_O_3_), is often related to the generation of free oxidizing radicals (ROS) within the intracellular compartments, termed as ferroptosis. Ferroptosis, distinct from the apoptotic cell death pathway, is generally characterized by the accumulation of iron species and lipid peroxidation (LPO), which can restrict the usual intrinsic apoptotic resistance of tumor cells. This characteristic has been applied to glioblastoma (GBM) therapy using biochemical species and compounds to induce LPO-mediated ferroptosis [33]. In this view, recent studies have demonstrated the potential use of the adjustable intrinsic enzyme-like activity of nanozymes related to the ferroptosis mechanism as an additional weapon against cancer [1,6,21,27,28,29,30,31,32]. The ferroptosis process can be associated with other cancer therapies, including surgery, hyperthermal therapy, chemotherapy, and radiotherapy, to augment the efficiency of killing cells in tumors. Surprisingly, despite being a fascinating research realm, it has been scarcely investigated because of its complexity and challenging characteristics [1,6,21,27,28,29,30,31,32].

Despite the undisputable success of nanomedicine in cancer research and technology, only 0.7% (median) of the nanoparticle dose is effectively delivered to a solid tumor. Moreover, only approximately 5% of anticancer drugs are projected to reach the clinical phase due to inappropriate pharmacokinetics or low drug efficacy [9,34]. Some researchers have claimed that nano-oncology is trapped by relying upon preclinical animal models due to a lack of essential fundamental knowledge of the biointerfaces involving complex nanomaterial–tissue interactions and events in the human body, from crossing biological barriers to their clearance from the body [15,35]. Additionally, the complexity of understanding the physical, structural, and biochemical aspects of the tumor microenvironment plays an important role in the cancer tumor growth. Thus, it is vital to elucidate more precisely how malignant cells can interact, transfer signals, and communicate with several supportive tumor-associated cells, including endothelial cells, fibroblasts, macrophages, and other immune cells. To avoid these complications and investigate the intricate mechanisms closely related to cancer progression, 3D spheroid and organoid models are usually the most preferable choices. These models can reproduce the stromal environment and multicellular structure found in a cancer tumor in vivo. Moreover, they offer more precise data regarding the tumor characteristics, cell–cell interactions, drug activity, and metabolic profile of cancer cells in comparison with oversimplified or unreliable approaches and unrepresentative animal models [9,34]. In addition, cancer biology research has been mostly performed relying on experimental designs involving 2D cultures; however, 2D cell culture practices have several restrictions.

Direct comparisons have evidenced that 3D tumor models simulate TMEs better than 2D cell cultures, as 2D methods fail to properly replicate the cell–cell and extracellular environment interactions [34,36]. Therefore, as a conventional 2D model based on monolayer cell cultures cannot precisely evaluate drug resistance, which may lead to misleading results, efforts have been made to develop new in vitro tumor models to represent the TME more accurately, such as 3D spheroid models [34,36,37].

Solid tumors in vivo usually exhibit key features, such as cell–cell signaling, cellular heterogeneity, growth kinetics, ECM interactions, and drug resistance, which can be effectively replicated in 3D spheroids [34,36]. Furthermore, recent studies have revealed biological processes unique to the human body that cannot be properly modeled in other animals, such as brain development, metabolism, and drug efficacy evaluations [34,38]. In this view, 3D spheroids and organoids can serve as an excellent complement to current therapeutic development strategies, bridging the gap between in vitro and in vivo research. They have the potential to significantly advance drug development by providing a more physiologically relevant platform for testing efficacy and toxicity [12,34,36,39].

Hence, here we hypothesize for the first time the design, synthesis, and comprehensive characterization of Co-doped iron oxide nanozymes (Co_x_Fe_3-x_O_4_, x = Co-content, x = 0.0, 0.1, 0.2, and 0.4) chemically functionalized by the carboxymethylcellulose (CMC) biopolymer. We render stable supramolecular colloidal nanoparticles that could be applied as anticancer nanotherapeutic agents for killing glioblastoma through a ferroptosis-based mechanism (i.e., endogenous biocatalytic activity) using a 3D spheroid model, which would be more precise than the usual 2D models in resembling the tumor microenvironment.

## 2. Experimental Procedure

### 2.1. Materials

Sodium carboxymethyl cellulose (CMC, degree of substitution: 0.7, average molecular mass: 90,000 Da), iron (II) sulfate heptahydrate (FeSO_4_·7H_2_O), iron (III) chloride hexahydrate (FeCl_3_·6H_2_O), cobalt (II) acetate tetrahydrate (Co(CH_3_COO)_2_·4H_2_O), ammonium hydroxide (NH_4_OH), citric acid, sodium phosphate dibasic, 3,3′, 5,5′ tetramethylbenzidine (TMB), (3-(4,5-dimethylthiazol-2yl-) 2,5-diphenyl tetrazolium bromide) (MTT), Triton™ X-100, 2′,7′-dichlorodihydrofluorescein diacetate (DCF-DA), tert-butyl hydrogen peroxide (TBHP), thiobarbituric acid (TBA), Bradford reagent, bovine serum albumin (BSA), and hydrochloric acid were supplied by Sigma-Aldrich (St. Louis, MO, USA). Hydrogen peroxide (H_2_O_2_) was provided by Merck (Darmstad, Germany). 5,5-dimethyl-1-pyrroline-N-oxide (DMPO) was supplied by Oakwood (Columbia, MO, USA). Dulbecco’s modified eagle medium (DMEN) and antibiotic-antimycotic solution were provided by Gibco (Grand Island, NE, USA). Fetal bovine serum (FBS) was provided by Cripion Biotecnologia Ltda. (Andradina, Brazil). Sodium dodecyl sulfate (SDS) was provided by LCG-Biotecnologia (Cotia, Brazil). Agarose low EEO (Electroendosmosis) was supplied by Fisher Scientific (Hampton, SC, USA). Tris-HCl buffer was provided by Labsynth Produtos para Labotatórios Ltda (Diadema, Brazil).

The previously mentioned chemicals were used without further purification. Deionized (DI) water (Millipore Simplicity^™^, Merck Millipore, Burlington, VT, USA) was used to prepare all the solutions with a resistivity of 18 MΩ·cm. The protocols and procedures were conducted at room temperature (RT, 25 ± 2 °C) unless specified otherwise.

### 2.2. Synthesis of MION and Co-MION Nanoparticles

Aqueous colloidal magnetic iron oxide nanoparticles (Fe_3_O_4_, MION) and the cobalt-doped nanoparticles (Co_x_Fe_3−x_O_4_, Cox-MION) were synthesized in alkaline conditions using NH_4_OH based on the coprecipitation method (Table 1). In brief, solutions were prepared by dissolving the iron salts (0.02 M of FeSO_4_ and 0.04 M of FeCl_3_) under vigorous stirring in the CMC solution (1.0% *w*/*v*, 200 mL) pre-heated to 40 ± 2 °C. Then, the solution was progressively heated up to 80 ± 2 °C using an inert atmosphere of nitrogen. In the sequence, 12 mL of NH_4_OH solution (25.0% *v*/*v*) was slowly poured into the reaction flask and slowly homogenized by stirring for 20 min. For the synthesis of cobalt-doped nanoparticles (Cox-MION), the amount of Fe(II) precursor used was partially replaced by Co(II), according to Table 1. Then, the colloidal solutions were cooled down to RT and dialyzed for 24 h against distilled water (cellulose membrane, cutoff of 12,000 Da, Sigma-Aldrich, St. Louis, MO, USA) under moderate stirring at room temperature and stored at 6 ± 2 °C. The concentrations of Cox-MION and metals (Fe and Co), the active species, were estimated based on the initial concentration of precursors added to the synthesis, the reactions involved, and the reaction yield of 90%.

### 2.3. MION and Co-MION Nanoconjugates—Morphological, Physicochemical, and Magnetic Characterization

Nanoparticle morphology and size distribution were analyzed at an accelerating voltage of 200 kV using transmission electron microscopy (TEM, Tecnai G2-20, FEI Company, Hillsboro, OR, USA). After dilution using DI water, samples for TEM analyses were prepared by placing droplets of the nanocolloidal suspensions onto holey carbon copper grids. The size distributions were acquired by measuring at least 50 nanoparticles randomly selected using the ImageJ software (National Institutes of Health, NIH, public domain).

X-ray diffraction (XRD, Cu-Kα radiation, PANalytical Empyrean diffractometer, Malvern, UK) was used to identify the crystalline structure of nanoparticles. The XRD diffraction patterns were recorded in the 2θ range from 3 to 90° with a step scan of 0.06° s^−1^. XRD analyses were performed using films obtained by casting method at 40 ± 1 °C.

X-ray photoelectron spectroscopy (XPS) measurements were performed with a monochromatic Mg-Kα X-ray excitation source (Amicus spectrometer, Kratos, Kanagawa, Japan). Binding energies were corrected based on the carbon 1 s signal (284.8 eV).

Fourier-transform infrared spectroscopy (FTIR) analysis was performed using transmission technique (Nicolet 6700, Thermo Fisher Scientific Inc., Waltham, MA, USA) from film samples obtained after casting and drying (40 °C/24 h) the nanoconjugates (wavenumber range: 400 to 4000 cm^−1^; scans: 64; and resolution: 2 cm^−1^).

Zeta potential (ZP) and dynamic light scattering (DLS) analyses were conducted (ZetaPlus, Brookhaven Instruments Corporation, Holtsville, USA) with a laser source with λ = 660 nm. All experiments were conducted at RT, and the light scattering was measured at 90°. At least ten (*n* ≥ 10) measurements were collected for each sample, and the average values were calculated.

Electron magnetic resonance (EMR) analysis was performed with an X-band electron paramagnetic resonance spectrometer (Magnettech, model MiniScope MS400, Freiberg, Germany) operating at 9.44 GHz, with a field modulation frequency of 100 kHz and a center field of 350 mT with a scan range of 500 mT, a scan time of 60 s, and 4096 integration points. EMR spectra were measured at RT after drying samples to reach a nanopowder consistency inside borosilicate tubes at 120 °C (Wilmad Labglass, Vineland, NJ, USA).

### 2.4. The Catalytic Activity of MION and Co-MION Nanozymes

The catalytic activity of the Cox-MION was tested by detecting the oxidation of chromophore 3,3′,5,5′-tetramethylbenzidine hydrochloride (TMB/TMB_ox_) by hydrogen peroxide (H_2_O_2_) substrate. Absorption measurements detected the TMB_ox_ oxidized species in a microplate reader at wavelength of λ = 655 nm (iMark™ Microplate Absorbance Reader, Bio-Rad^®^, Hercules, CA, USA).

The assay of Cox-MION was prepared by adding 10 µL of nanoparticle (600 µg mL^−1^) and 20 µL of TMB solution (0.4 mM in DI water) to a microplate well. In the sequence, 120 μL of phosphate-citrate buffer (pH = 5.0 ± 0.2) and 50 μL of H_2_O_2_ solution (stock solution = 0.2 M in DI water) were added in each well (V_total_ = 200 μL) (replicates, *n* = 5).

To evaluate the mechanisms of catalytic activity of nanoparticles, electron paramagnetic resonance (EPR) experiments amalgamated with the spin trap methodology were performed on a MiniScope MS400 spectrometer (Magnettetech, Freiberg, Germany) operating at X-band (microwave frequency of ~9.4 GHz). Experimental parameters used in the measurements were as follows: 10 mW of microwave power, a modulation field of 100 kHz with an amplitude of 0.2 mT, a center field of 337 mT with a scan range of 10 mT, a scan time of 60 s, and 4096 integration points. All EPR spectra were measured at RT. The spin trap used was DMPO (5,5-dimethyl-1-pyrroline-N-oxide).

Measurements were performed using hydrogen peroxide in colloidal aqueous solutions of MION and Co40-MION nanocatalysts at pH = 5.0 ± 0.2 (citrate-phosphate buffer). Aqueous stock solutions of MION and Co40-MION (3.1 mg mL^−1^ of iron-based oxide) samples were tested at three pH values. The aliquots used to obtain the EPR spectra were prepared using 50 μL of iron oxide solutions (72.5 μL of the Cox-MION suspension to 828 μL of buffer solution), 100 μL of the spin trap solution (20 mg of DMPO in 500 μL of buffer solution), 20 μL of the H_2_O_2_ solution (10.2 μL of peroxide in 490 μL of buffer), and 30 μL of the buffer solution, resulting in a final volume of 200 μL. Then, the solutions were placed into glass capillaries with a volume of 50 μL and inserted into a quartz tube to carry out the EPR measurements at predefined times.

### 2.5. Biological Tests

Biological tests were performed using glioblastoma cancer cells (U87, American Type Culture Collection—ATCC^®^ HTB-14™) that were purchased from the Brazilian Cell Repository (Banco de Células do Rio de Janeiro: BCRJ, Rio de Janeiro, Brazil; cell line authentication molecular technique, Short Tandem Repeat (STR) DNA; quality assurance based on the international standard NBR ISO/IEC 17025:2005).

U87 cells (passage 63) were cultured in DMEM with 10% FBS and antibiotic-antimycotic solution (penicillin G sodium, 10 units mL^−1^; streptomycin sulfate, 10 mg mL^−1^; and amphotericin B, 0.025 mg mL^−1^) in a humidified atmosphere of 5% CO_2_ at 37 °C.

All biological tests were performed according to ISO 10993-5:2009/(R)2014 (Biological evaluation of medical devices: Tests for in vitro cytotoxicity).

Statistical significance was evaluated based on one-way ANOVA followed by Bonferroni’s method. A confidence level of α < 0.05 was considered statistically significant.

#### 2.5.1. 2D Cell Culture Bioassays

##### Cell Viability

To assess the preliminary nanocatalytic therapeutic efficiency in vitro, the cellular cytotoxicity of nanosystems was tested by MTT (3-(4,5-dimethylthiazol-2yl-) 2,5-diphenyl tetrazolium bromide) protocol, as previously reported by our group [12,24]. Briefly, U87 cells were plated (1 × 10^5^ cells/well) in 96-well plates, and the cell population was synchronized by nutrient deprivation for 24 h (DMEM medium without FBS). Then, the media volume was suctioned, replaced with a DMEM medium containing 10% FBS, and incubated for 24 h. Then, cells were treated with 1.8 × 10^−4^–60 μg mL^−1^ of MION and Co40-MION suspensions (based on Fe or Fe + Co active agent) for 24 h. Then, the total volume in each well was aspirated and replaced with 60 µL of culture media containing serum. The MTT reagent (5 mg mL^−1^) was added to each well and incubated for 4 h in an oven at 37 °C and an atmosphere of 5% CO_2_. Next, 40 µL of SDS solution/4% HCl was placed in each well and incubated for 16 h in an oven at 37 °C with an atmosphere of 5% CO_2_. Then, 100 µL from each well was aspirated and transferred to a similar blank 96-well plate. The absorbance of samples was evaluated at a wavelength of λ = 595 nm, using a Varioskan Lux Microplate Absorbance Reader (Thermo Fisher Scientific Inc., Waltham, MA, USA). The percentage of cell viability response was calculated after blank corrections, according to Equation (1), where the values of the control group were set to 100% cell viability. Results were calculated based on the mean and SD of six replicates (*n* = 6).

(1) $$\mathrm{Cell}\;\mathrm{viability}=\frac{\mathrm{Absorbance}\;\mathrm{of}\;\mathrm{sample}\;\mathrm{and}\;\mathrm{cells}}{\mathrm{Absorbance}\;\mathrm{of}\;\mathrm{control}}\;\times \;100$$

##### Formation of Intracellular Reactive Oxygen Species (ROS)

The relative intracellular concentration of ROS was measured based on a 2′,7′-dichlorodihydrofluorescein diacetate experiment (DCF-DA), as previously reported by our group [3,24]. In brief, DCF-DA is a cell-permeable non-fluorescent reagent that is deacetylated by cellular esterases to the non-fluorescent dichlorodihydrofluorescein (DCFH) after cell uptake. In the presence of intracellular H_2_O_2_, hydroxyl radicals, and peroxyl radicals, DCFH is oxidized to the green fluorescent 2′,7′-dichlorofluorescein (DCF), therefore allowing us to estimate the ROS species based on photoluminescence spectroscopy (PL) [3,40].

U87 cells (1 × 10^4^ cells/well on 96-well plates) were incubated with 100 µL of 2′,7′-DCF-DA solution at 100 µM (diluted in DMEM medium) for 40 min in an oven at 37 °C and atmosphere of 5% CO_2_. After this period, the medium volume was aspirated, and U87 cells were exposed to 100 µL of MION and Co40-MION (0.6 and 6 μg mL^−1^ of Fe or Fe + Co). Cells were also incubated with only DCF-DA (negative control) and with tert-butyl hydrogen peroxide (TBHP, 5.0 μM in water, positive control). After 30 min and 120 min incubation, at 37 °C/5% CO_2_, the fluorescence intensity of DCF was measured using Varioskan™ LUX multimode microplate reader (Thermo Fisher Scientific Inc., Waltham, USA; λ_excitation_ = 488 nm, and λ_emission_ = 528 nm). When comparing cells, the PL intensity values were expressed as a percentage of fluorescence intensity relative to the positive control wells (100%). Results were calculated based on the mean and standard deviation (SD) of three replicates (*n* = 3).

##### Lipid Peroxidation

Lipid peroxidation was analyzed using the thiobarbituric acid method (TBA test) to determine malondialdehyde (MDA) by UV–Vis spectroscopy after 24 h of contact with the nanozymes as previously reported by our group [3]. In brief, U87 cells were plated (2 × 10^4^ cells/well) in 6-well plates. Then, MION and Co40-MION suspensions were added to individual wells at a concentration of 6 μg mL^−1^ (of Fe or Fe + Co). Control samples were designed as follows: control (cell culture with DMEM and 10% FBS); positive control (cell culture with DMEM, 10% FBS, and 5 μM of TBHP); and negative control (cell culture with DMEM, 10% FBS, and 1 mg mL^−1^ chips of sterile polypropylene Eppendorf^®^). After incubating for 24 h, cells were washed two times with Tris–HCl buffer (400 mM, pH 7.3) and treated with 1 mL of a solution containing TBA (0.4%, *w/v*), SDS (0.5%, *w/v*), and acetic acid (5%, *v/v*) at pH of 3.5. Cells were scraped and incubated at 95 °C (Termomix, Eppendorf F1.5, Hamburg, Germany). After 60 min, the reaction was stopped in ice bath for 5 min. Then, 300 µL was transferred to a blank 96-well plate, and the absorbance was measured using a Varioskan™ LUX multimode microplate reader (Thermo Fisher Scientific Inc., Waltham, USA) at λ = 532 nm. The results were calculated as nmol of MDA-TBA/mg of cellular protein using 156 mM^−1^ cm^−1^ as a molar extinction coefficient of MDA-TBA. Proteins extracted from cells were calculated using the Bradford method with bovine serum albumin as reference material. Data and results were presented as the mean and SD of three replicates (*n* = 3).

#### 2.5.2. 3D Cell Culture Tests (Tumor Spheroids)

##### Tumor Spheroids’ Generation and Treatment

Spheroids were generated in vitro using the low attachment plate method according to the protocol reported by Henrique et al. [41,42]. In brief, 96-well plate culture dishes were coated using a solution of 2% *w/v* agarose dissolved in double distilled water and dried for 30 min inside the laminar flow. After gelling, the thin agarose film was sterilized by UV radiation for 30 min (at 10 cm from the source, lamp power of 30 W, and wavelength of 254 nm). In the sequence, the culture medium DMEM with 10% FBS was added together with the U87 cells previously cultivated as monolayers (1.2 × 10^4^ cells by well). The cells were cultured using a humidified atmosphere with 5% CO_2_ at 37 °C. The culture medium was replaced every 3 days and observed every day under an inverted microscope (TMS, Nikon, Tokyo, Japan). Tumor spheroid formation was confirmed visually ten days later [42].

**Figure 1:**
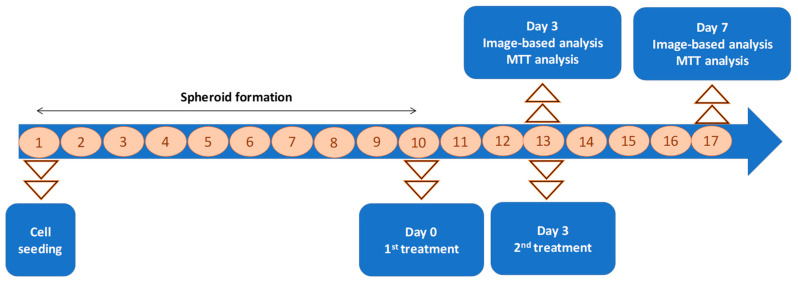
Timeline of in vitro generation of tumor spheroids (3D cell culture) and treatment applications.

After the formation of spheroids (10th day), they were treated with MION, Co40-MION, and DOX at a concentration of the active agents (Fe + Co or DOX) of 0.6, 6, and 60 μg mL^−1^ (Day 0). After 3 days (the 13th day of the experiment, Day 3), the morphological features and cell viability of spheroids were evaluated, and the treatment administration was repeated. The morphological and cell viability tests were performed after 7 days of the first administration (4 days after the repeated administration, the 17th day of the experiment, Day 7). The first set of analysis was identified as 3 days (or the third day of treatment) and the second as 7 days (or the seventh day of treatment). The timeline in Figure 1 summarizes these data.

##### Tumor Spheroid Size Evaluation

After the formation of the spheroids, they were analyzed and photographed (Nikon ECLIPSE microscope) before treatment (Day 0) and on the third (Day 3) and seventh days (Day 7) of treatment.

The spheroid dimensions were evaluated by measuring their diameters using Image-J software (NIH, public domain). Data were presented as the mean and the standard error (SE) of three replicates (*n* = 3).

##### Evaluation of Cell Viability of 3D Tumor Spheroid

Cell viability was detected using MTT assay, following the modified protocol by Dilnawaz et al. [43]. Triplicates of spheroids were used for each treatment (MION, Co40-MION, and DOX) and untreated control condition. On the third and seventh days, the spheroids were aspirated, trypsinized, and converted into separate cells in suspension. The cells were centrifuged at 1400 rpm for approximately 5 min, and the pellets were resuspended, treated with the MTT reagent (5 mg mL^−1^, >98%, Sigma-Aldrich, St. Louis, MO, USA), and incubated in an oven at 37 °C and 5% CO_2_ for 4 h.

After this period, the cells were centrifuged (1400 rpm, 5 min), the MTT solution was aspirated, and the pellets were resuspended in 100 µL of isopropanol/HCl 4% solution to dissolve the formazan crystals. Then, a volume of 100 µL was transferred to a similar blank 96-well plate, and the cell viability was calculated as described in Section “*Cell Viability*”. using Equation (1).

## 3. Results and Discussion

### 3.1. Characterization of MION and Co-MION Nanozymes

**Figure 2:**
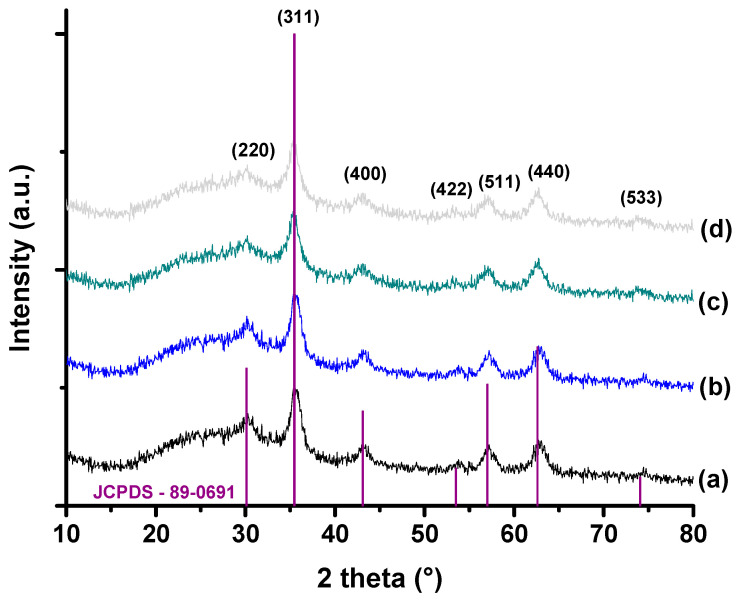
Diffraction patterns (XRD) of Cox-MION (x = 0, 10, 20, and 40%) samples ((a) MION, (b) Co10-MION, (c) Co20-MION, and (d) Co40-MION); data on the bottom are a reference pattern of standard Fe_3_O_4_ (JCPDS-89-0691).

The XRD patterns (Figure 2) of Cox-MION (x% = 0, 10, 20, and 40%mol of cobalt to Fe^2+^) nanocolloids are well-matched magnetites with a single-phase inverse spinel structure (JCPDS—89-0691) which was expected based on the synthesis procedure (the use of Fe^2+^ and Fe^3+^ precursors with a N_2_ atmosphere and alkaline pH). Peaks were observed at 2θ (±0.2) = 30.1°, 43.1°, 35.5°, 53.5°, 57.0°, 62.6°, and 74.1° which are associated with the plane orientations (220), (311), (400), (422), (511), (440), and (533), respectively. The crystallite sizes of the nanoparticles measured by Scherrer’s equation were about 5–7 nm (Table S1). Moreover, based on the XRD profiles, the cobalt doping did not significantly affect the crystalline pattern of the nanoparticles within the concentration range investigated. For all the nanozymes profiles, the broad diffused hump for 2θ between 20 and 25° is associated with the CMC biomacromolecular ligand with an amorphous pattern.

As heterogeneous catalytic processes are highly dependent on the solid–liquid interfaces, the XPS analyses were executed as a highly sensitive surface characterization technique for quantifying the chemical states of surface atoms to confirm nanosized magnetite formation.

**Figure 3:**
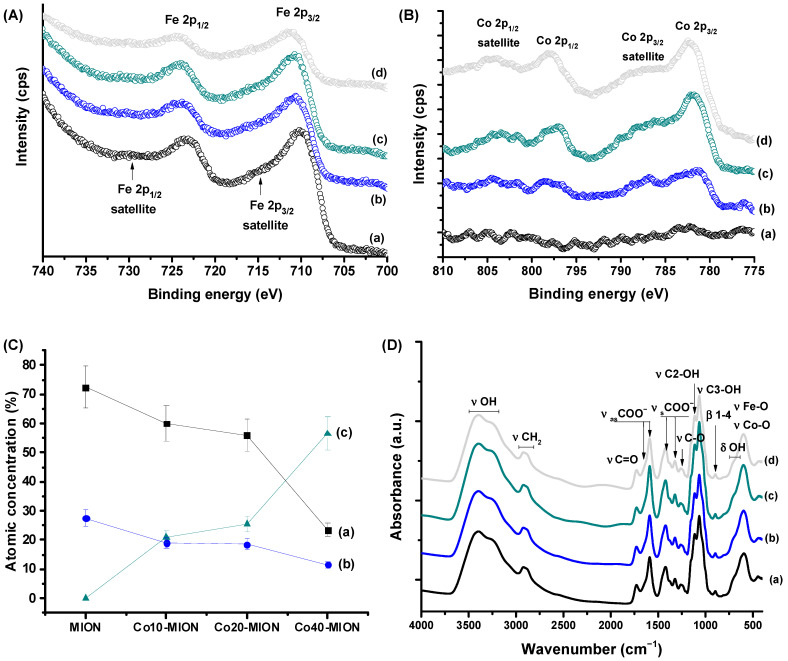
XPS spectra of (**A**) Fe 2p and (**B**) Co 2p regions for Cox-MION nanoconjugates: (a) MION, (b) Co10-MION, (c) Co20-MION, and (d) Co40-MION. (**C**) Quantitative analysis of the distribution of (a) Fe(II), (b) Fe(III), and (c) Co(II) species at the surface of Cox-MION nanoconjugates. (**D**) FTIR spectra of (a) MION, (b) Co10-MION, (c) Co20-MION, and (d) Co40-MION.

Figure 3A,B show the XPS spectra of Cox-MION nanoconjugates obtained for the Fe 2p and Co 2p regions, respectively. The high-resolution XPS spectra of the MION iron oxide nanoparticles (Figure 3A) show two prominent peaks at 710.4 eV (Fe 2p_3/2_) and 723.8 eV (Fe 2p_1/2_) with respective satellite peaks, which are related to the Fe 2p transitions in magnetite nanoparticles overlapping the contributions of both iron cations, Fe(II) and Fe(III). Additionally, the absence of a satellite peak nearby at 719 eV demonstrates both that iron species (i.e., Fe^2+^ and Fe^3+^) are present in the lattice and the formation of magnetite iron oxide [24,44,45]. As the cobalt content increased, the peak intensities of the Fe 2p gradually weakened, followed by the relative broadening of their peak shapes, demonstrating a comparative decrease in the Fe species in the samples due to Co substitution. Simultaneously, the Co 2p_3/2_ (781.5–782.2 eV) and Co 2p_1/2_ (797.3–798.0 eV) peaks as well as their respective satellite peaks became stronger and sharper with the increase in Co (Figure 3B). In addition, the presence of Co 2p_3/2_ centered at a distance of 5–6 eV from the Co 2p_3/2_ component showed the occurrence of divalent cobalt cations (Co^2+^) [24,44,46]. The linear regression of the curves of atomic concentration (obtained from the peak area, Figure S1) of Co^2+^ and Fe^2+^ as a function of the cobalt content (R^2^ ≥ 0.94) demonstrated that these changes in intensity were mostly associated with doping with cobalt.

The FTIR spectra (Figure 3D) presented the characteristic peaks of the CMC ligand [6,17]. The bands in the 3500–3200 cm^−1^ range are associated with stretching vibrations of free hydroxyl groups and hydrogen-bonded CMC molecules. The bands at 2950–2850 cm^−1^ are assigned to νCH_2_. The ν_as_COO^−^ and ν_s_COO^−^ regions of carboxylate species were observed at 1650/1590 cm^−1^ and 1420/1320 cm^−1^. Additionally, bands related to the protonated carboxylic group were detected at 1730 cm^−1^ (νC=O) and 1250 cm^−1^ (νC-O). In addition, stretching bands of the alcohol groups (νC-OH, 1110/1060 cm^−1^) and glycoside bonds (β1-4, 890 cm^−1^), as well as bending vibrations of the hydroxyl groups (βO-H, 710–600 cm^−1^) were observed. The bands centered at 620–590 cm^−1^ revealed the presence of iron-oxide-based nanoparticles, which are associated with the νFe-O and νCo-O vibrations of the oxygen–metal ion complex [3,47]. Additionally, the occurrence of two carboxylate bands indicated the formation of two types of coordination in the CMC–metal complexes, where the wavenumber differences (Δ(ν_as_COO^−^)–(ν_s_COO^−^)) suggest the formation of monodentate and bidentate binding [48,49].

**Figure 4:**
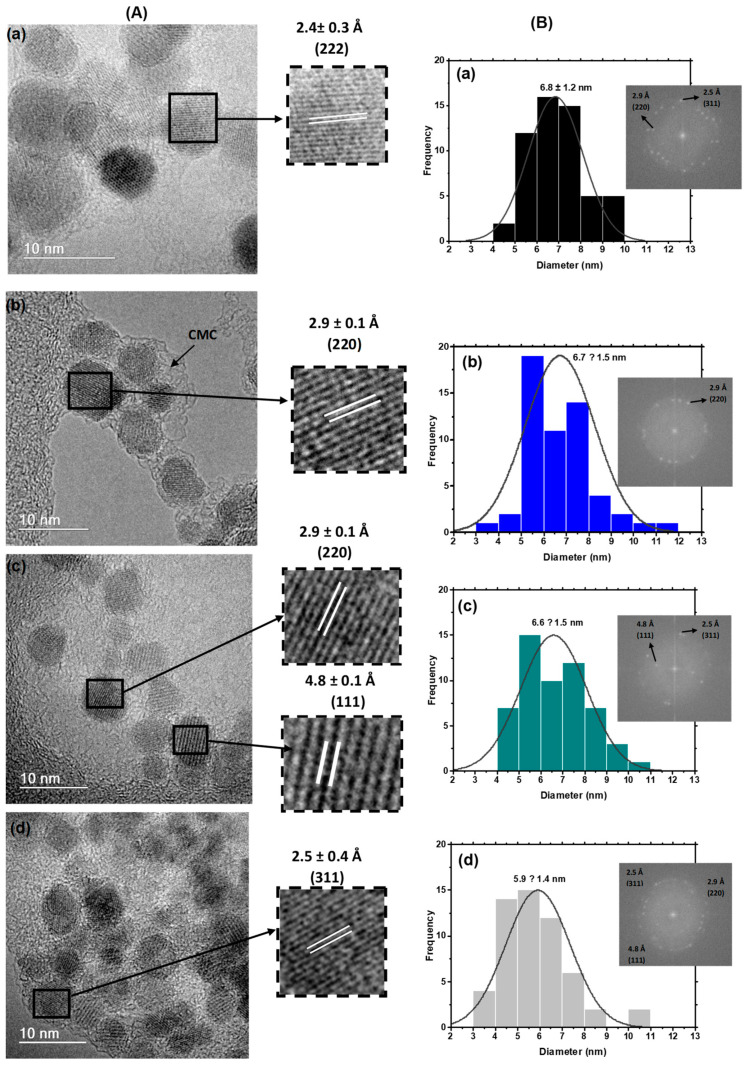
Morphological and structural characterization of Cox-MION nanozymes ((**a**) MION, (**b**) Co10-MION, (**c**) Co20-MION, and (**d**) Co40-MION): (**A**) TEM images (inset, interplanar lattice fringes with interplanar distances); and (**B**) Histogram of the size distribution (inset, SAED patterns).

The TEM images indicated that the magnetite nanoparticles and magnetite doped with up to 40% cobalt were produced with a predominantly spherical morphology (Figure 4A). The light contrast could also be observed around the nanoparticles due to the CMC polymer used as the nucleation/stabilizing agent in the dry state. The average size (Figure 4B) ranged from 6.8 ± 1.2 nm (x = 0%) to 5.9 ± 1.4 nm (x = 40%), indicating a moderate decrease in the average size of 13% with the maximum cobalt content. This trend was confirmed by one-way ANOVA and Bonferroni’s statistical analyses (*p* < 0.05) (Figure S2).

This can be ascribed to the partial replacement of Fe^2+^ in the octahedral B sites of the magnetite nanocrystals with an ionic radius = 0.77 Å by Co^2+^ which exhibits a smaller ionic radius (0.74 Å), causing the “shrinkage” of the unit cell [50,51].

The nanoparticles’ diameters based on the TEM analysis are comparable to the average crystallite sizes measured by Scherrer’s equation (Table S1), suggesting that the nanoparticles are single nanocrystals rather than polycrystalline [52].

High-resolution transmission electron microscopy imaging (HRTEM) was also used to investigate the crystallinity of MION and Cox-MION nanoparticles. The presence of interference fringes with average distances (d ± 0.1 Å) of 2.5 Å, 2.9 Å, and 4.8 Å (Figure 4A, inset) was associated with the interatomic distances of (220), (311), and (111) planes of magnetite, respectively. Moreover, the selected area’s electron diffraction patterns (SAED, inset in Figure 4B) comprised concentric diffraction rings. These images were indexed as (220), (311), (400), (511), and (440) planes consistent with the cubic spinel structure of ferrites, with d-spacings (d ± 0.1 Å) of 4.8 Å, 2.9 Å, and 2.50 Å, respectively. These results confirmed the crystallinity of the iron oxide nanoparticles and endorsed the previous findings of the XRD analysis.

ZP and DLS were performed to evaluate the nanoconjugate’s surface chemistry and colloidal properties (Figure S3A). The ZP values at a pH of 7.2 were reasonably stable, approximately −50 mV, with no detectable dependence on the cobalt content. CMC is a pH-sensitive multifunctional (R-COOH/COO^−^ and OH) polysaccharide due to the deprotonation of the carboxylic acid groups (-R-COOH) to anionic carboxylates (R-COO^−^). Above the pKa value (~4.6), the CMC is fully deprotonated (-COO^−^), rendering negative charges for the polymer chains, and when the pH is reduced below the pKa, the carboxylate groups become gradually protonated, forming -R-COOH species. The CMC polymer is expected to act as a stabilizing ligand for iron-oxide-based nanoparticles after chemisorption via the coordination bonding of the carboxylate groups with iron and cobalt cations at the surface of the nanoparticles (COO–Metal). As shown in Figure S3B, the CMC polymers remain anionic even at a pH of approximately 3.5 (−20 mV) and become more negative above this pH value. In this sense, this ZP resulting at a neutral pH agrees with the negative charge associated with the deprotonated carboxylic groups (COO^−^) of the CMC polymer that electrostatically stabilize the colloidal systems with limited effect from the cation (Fe/Co) of the inorganic core. Nonetheless, steric hindrance from polymer chains may also be present to a lower extent and cannot be discarded. A reduction in the modular value of the ZP of Cox-MION could also be observed compared to that of the pure polymer at the same pH. This effect was expected as the chemical coordination of the COO^−^ groups of the CMC with the cations of the nanoparticle surface causes a relative depletion of total carboxylate groups from the polymer chain. In addition, changes in pH are not expected to significantly affect nanoparticle stabilization because, when the ZP > |30| mV, the electrostatic stabilization will dominate, and when ZP < |30| mV, steric hindrance will guarantee the system’s stability. When considering changes in the ionic strength, as expected in biological media, the screening effect of positive ions on the negative charges of the polymer is expected to reduce the influence of the repulsive Coulomb forces acting between the charged chains. Still, due to steric hindrance from the polymer, the iron oxide inorganic core is expected to remain unchanged.

**Figure 5:**
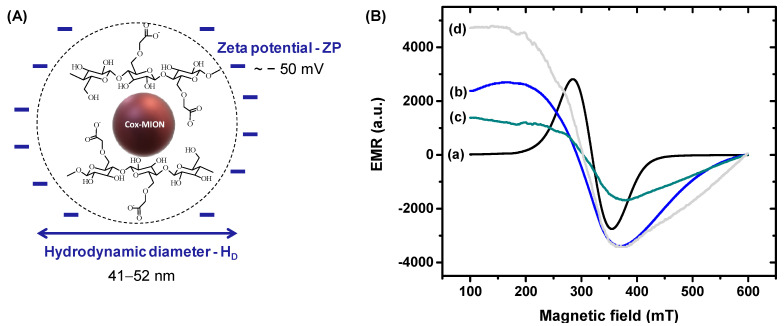
(**A**) Schematic representation of supramolecular nanostructure of Cox-MION nanozymes. (**B**) EMR spectra from: (a) MION, (b) Co10-MION, (c) Co20-MION, and (d) Co40-MION measured at room temperature (microwave frequency of 9.44 GHz).

Regarding the DLS studies at a pH of 7.2, the hydrodynamic diameter (H_D_) of the polymer-coated nanosystems (Cox-MION) varied from 41 nm up to 52 nm, indicating that the CMC is first and foremost responsible for the colloidal dimension (“size”) of the nanoconjugate in the aqueous medium. Figure 5A summarizes a schematic representation of the supramolecular nanostructure of the nanozyme conjugates composed of an inorganic core (Co-MION) and polymer shell forming water-stable nanocolloids.

We used electron magnetic resonance (EMR) spectroscopy to investigate the magnetic properties of the nanoconjugates. The MION nanoparticles (Figure 5B(a)) showed characteristic EMR signals when evaluated at room temperature (T = 300 K) for superparamagnetic nanoferrites [53]. However, changes in the resonance lines (Figure 5B(b–d)), such as broad and asymmetric shape lines with lower intensities, were observed with the increase in the cobalt content, suggesting a progressive transition from superparamagnetic to ferrimagnetic, which could be associated with an increase in the magnetocrystalline anisotropy of magnetite nanoparticles [54].

Although not directly focused on in this study, these superparamagnetic ferrofluids (e.g., Fe_3_O_4_) have been of great interest recently due to their unique magnetic properties. They can be used in cancer nanomedicine and other biomedical applications, including magnetically guided target drug delivery, magnetic hyperthermia processes, cell-, DNA-, and protein-separation, and magnetic resonance imaging (MRI) for diagnosis [1,3,55,56]. Thus, besides the biocatalytic activity of these nanoconjugates investigated in our research, their magnetic properties may be explored in future cancer nanotheranostics studies.

### 3.2. Nanozymes—Enzymes Mimicking Catalytic Activity—Acellular In Vitro

#### 3.2.1. Oxidation of TMB by Reactive Oxygen Species (ROS)

The catalytic functionality of the magnetite and Co-doped nanozymes was primarily assessed by the oxidation of 3,3′,5,5′-tetramethylbenzidine hydrochloride (TMB) as a well-established colored substrate for similar systems mimicking natural enzymes (e.g., horseradish peroxidase, HRP). Reactive oxygen species (ROS), such as hydroxyl (•OH) and hydroperoxyl (•HO_2_) radicals, are produced from hydrogen peroxide under catalysis by iron-oxide-based nanoparticles based on a Fenton-like reaction according to Equations (2) and (3). The generated hydroxyl radicals (•OH) have a much stronger oxidation capacity (E° = 2.80 V) than that of hydrogen peroxide H_2_O_2_ (E° = 1.77 V) and hydroperoxyl (•HO_2_, E° = 1.65 V) [57]. These radicals oxidize TMB to TMB_ox_ species, which were detected using the blue-colored absorbance measured by UV–vis spectroscopy.

Fe(II)_(surface)_ + H_2_O_2(ads)_ → Fe(III)_(surface)_ + •OH + HO^−^ (2)

Fe(III)_(surface)_ + H_2_O_2(ads)_ → Fe(II)_(surface)_ + •HO_2_ + H^+^ (3)

**Figure 6:**
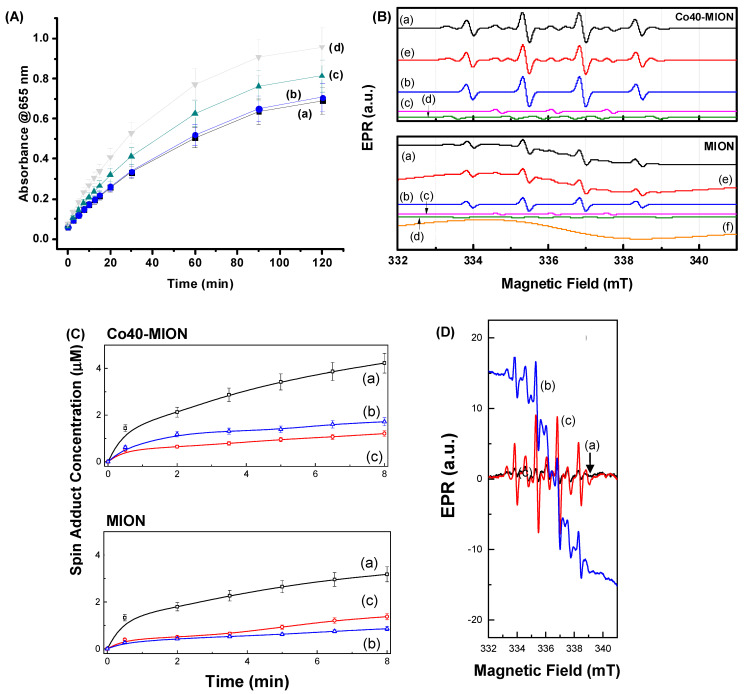
(**A**) Effect of cobalt doping in the catalytic oxidation of TMB ((a) MION, (b) Co10-MION, (c) Co20-MION, and (d) Co40-MION nanoconjugates. (**B**) Measured EPR spectra (a) for Co40-MION and MION samples obtained after adding and interacting (1 min) hydrogen peroxide (~0.06%) with the aqueous solutions containing DMPO spin trap. Calculated EPR spectra of three different spin adducts: (b) DMPO/•OH, (c) DMPO*, and (d) DMPO/•CH(OH)CH_3_. Line (e) corresponds to the sum of the different contributions, and line (f) corresponds to a superparamagnetic contribution to the EPR spectrum of the MION sample. (**C**) Kinetics of spin adduct concentration for Co40-MION and MION samples as a function of the reaction time with H_2_O_2_ (~0.06%): (a) •OH radical, (b) •CH(OH)CH_3_ radical, and (c) DMPO* radical adduct. (**D**) EPR spectra of DMPO adducts after interaction of H_2_O_2_ (~0.06%, 8 min) with and without catalyst samples: (a) without nanozyme, (b) MION, and (c) Co40-MION.

The evolution of the TMB_ox_ absorbance spectra with time upon the injection of H_2_O_2_ (50 mM) into the Cox-MION suspensions (citrate-phosphate buffer, pH 5.0, Cox-MION concentration of 30 μg mL^−1^) is presented in Figure 6A. The pH of 5.0 was selected due to the acidic microenvironment of tumors (pH of 5.6 to 6.8), compared to that of healthy cells and tissues (pH of 7.2–7.4) [58]. These results evidenced the relevant booster effect of incorporating cobalt into the nanocatalysts, for which much more progress of the chromogenic TMB_ox_ was observed with Co40-MION. This effect was ascribed to the substitution of Fe^2+^ by Co^2+^ into Fe_3_O_4_ nanostructures, improving their activity in the Fenton reaction and promoting a higher decomposition rate of H_2_O_2_ into •OH free radicals [59,60]. This can be explained by comparing the redox potential of Co^3+^/Co^2+^ (E° = 1.81 V, Equation (4)), which is higher than that of Fe^3+^/Fe^2+^ (E° = 0.77 V, Equation (5)). Thus, the generation of Co^2+^ in the medium would be thermodynamically favorable compared to ferrous ions (Equation (6)). Consequently, this would increase the H_2_O_2_ decomposition and produce more oxidizing radicals.

Co(III)_(aq)_ + e^−^ → Co(II)_(aq)_       E° = 1.81 V (4)

Fe(III)_(aq)_ + e^−^ → Fe(II)_(aq)_       E° = 0.77 V (5)

Fe(II)_(aq)_ + Co(III)_(aq)_ → Fe(III)_(aq)_ + Co(II)_(aq)_       E° = 1.04 V (6)

#### 3.2.2. Spin-Trapping EPR Experiments—Mechanisms of ROS Generation

To confirm the mechanisms proposed in the previous sections that associated the catalytic behavior of Cox-MION with the formation of the highly active hydroxyl radicals based on a Fenton-like reaction, the identification of ROS generated by hydrogen peroxide (H_2_O_2_) over MION and Co40-MION was performed using the DMPO (5,5-dimethyl-1-pyrroline-N-oxide) spin trap. The EPR spectra obtained after adding H_2_O_2_ and allowing it to interact for 1 min are shown in Figure 6B for Co40-MION and MION. The generation kinetics of the spin adducts was evaluated with a reaction time at pH = 5, which is often associated with the tumor microenvironment, as previously discussed [58]. The generated spin adduct EPR spectra (Figure 6B, line a) are very similar for both samples and consist of the superposition of three spin adducts: DMPO/•OH (line b), DMPO* (line c), and DMPO/•CH(OH)CH_3_ (line d). The spin adducts were identified by spectra calculations using the Easyspin^@^ software. The sum of the three adduct contributions (line e) matched well with the measured EPR spectrum (Figure 6B, line a). A broad superparamagnetic contribution to the EPR spectrum for the MION sample was also noticed (Figure 6B, line f).

The DMPO/•OH spin adduct (Figure 6B, line b) is characterized by the interaction of an electronic spin *S* = ½ with a nuclear spin *I* = 1 for a ^14^N nucleus (99.6%) and with an *I* = ½ for a hydrogen atom (100%) in the molecular β position (a_N_ = a_H(β)_ = 1.50 mT) [61].

The DMPO spin trap is suitable for directly capturing hydroxyl radicals in aqueous solutions. Adding H_2_O_2_ to an aqueous solution containing iron oxide nanoparticles, Fenton or Fenton-like chemical reactions are expected to form mainly hydroxyl radicals. However, superoxide radical species may also be formed in Fenton-like reactions. In this case, the detection of the DMPO/•OH adduct can be associated with the conversion of the DMPO/•O_2_^−^ spin adduct, which only has a lifetime of milliseconds in water. Therefore, such an indirect chemical reaction with the superoxide radicals cannot be ruled out in the analyzed reaction. However, the latter should be more effective in the presence of iron oxide nanoparticles containing predominantly Fe^3+^ species.

The DMPO* spin adduct (Figure 6B, lines c) is characterized by the hyperfine interaction constant a_N_ = 1.47 mT between the nuclear spin *I* of a ^14^N nucleus with an electronic spin *S* = ½. Several authors have associated this EPR signal with the degradation of the DMPO molecule, which is not typical of ROS capture [62]. In contrast, other authors have associated it with the formation of a dimerization of two spin adducts after the capture of the •OH radical by two molecules of DMPO (DMPO-OH-DMPO) [63]. Finally, the DMPO/•CH(OH)CH_3_ adduct (Figure 6B, lines d) is characterized by the interaction of an electronic spin *S* = ½ with a nuclear spin I for a ^14^N nucleus (a_N_ = 1.55 mT) and with the hydrogen atom in the molecular β position (a_H(β)_ = 2.26 mT) [64]. This latter spin adduct is due to an organic molecule in the medium; therefore, it should originate from the biopolymer, i.e., the carboxymethyl cellulose used to prepare the MION and Co40-MION samples.

The first supposition reached by observing the generation kinetics of Figure 6C is that the dominant reactive species in the entire process is the •OH radical, directly formed or indirectly formed by the conversion of the DMPO/•O_2_^−^ adduct. The two samples, MION and Co40-MION, show similar generation kinetics, highlighting that cobalt could, in this case, play the role of the iron ion (most likely in the (2+) valence state) in the formation of ROS. The results indicated that the •OH radical is the dominant species responsible for the oxidation of the TMB (Figure 6A), detected in a much higher concentration via EPR. In addition, the experiment was repeated to separate the contribution of H_2_O_2_ alone without the catalysts (Figure 6D(a)) in the formation of ROS. In the presence of MION (Figure 6D(b)) and Co40-MION (Figure 6D(c)), the catalytic effect of iron oxide nanozymes was evidenced.

### 3.3. Biological Tests

#### 3.3.1. 2D Cell Culture Bioassays

**Figure 7:**
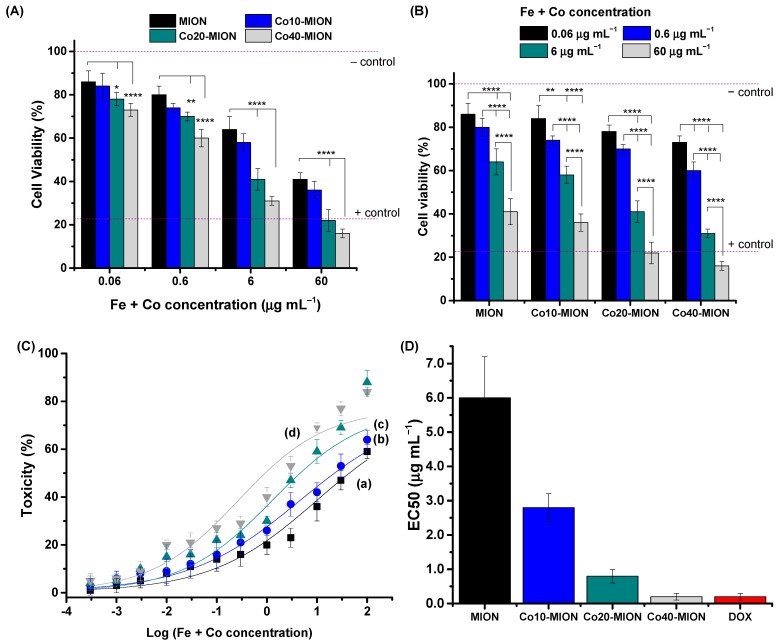
Comparative MTT of U87 cells incubated for 24 h with Cox-MION at Fe + Co concentrations of 0.06, 0.6, 6, and 60 μg mL^−1^: (**A**) Effect of cobalt content in nanozyme (statistical analysis related to MION at different concentrations of nanozyme); and (**B**) Effect of nanozyme dose (statistical analysis comparing the effect of concentration at each Cox-MION system). (**C**) Dose–response curves for Cox-MION. (**D**) EC-50 analysis (reference of anticancer chemotherapeutic DOX [1]. Statistical analyses of ‘one-way’ ANOVA, Bonferroni, and multiple comparisons: **** = *p* < 0.0001, ** = *p* < 0.01, and * = *p* < 0.05 (*n* = 5).

An MTT (3-(4,5-dimethylthiazol-2-yl)-2,5-diphenyl tetrazolium bromide) bioassay is one of the most regular tests to screen the effect of compounds for potential anticancer drug applications in cancer 2D cell culture [65]. Therefore, this study presents the results of the cell viability response of an MTT in vitro assay of the nanozymes for GBM cancer cells in Figure 7.

The cell viability results evidenced the higher cytotoxicity of Co-doped nanoconjugates (Figure 7A), with greater lethality with an increase in Co content, for all the concentrations investigated. A significant reduction in cell viability responses at the maximum cobalt content (x = 40%) compared to those of undoped nanozyme (x = 0%) was observed, with 22% at the lower concentration (0.06 µg mL^−1^) and higher than 40% for the other concentrations.

Moreover, the MTT results evidenced a reduction in cell viability responses by increasing the concentration of nanozymes, demonstrating that cell viability is concentration-dependent (Figure 7B). At lower concentrations (0.06 µg mL^−1^), the Cox-MIONs were non-toxic, according to the international standard ISO 10933-5 (cell viability > 70%). Co20-MION and Co40-MION showed crescent cytotoxicity from 0.6 µg mL^−1^ and MION and Co10-MION from 6 µg mL^−1^.

To compare the killing activity of Co-based nanozymes with doxorubicin (DOX), which has often been adopted as a model anticancer drug, the half-maximal effective concentrations (EC-50) of nanozymes were obtained for the U87 cell line incubated for 24 h with different concentrations of the nanozymes (Figure 7C). The data were fitted using the general equation for a sigmoidal model of dose–response curves (Toxicity versus log(Fe + Co) concentration, R^2^ ≥ 0.93), and the EC-50 values are presented in Figure 7D. A remarkable decrease in the EC-50 parameter was observed with the increase in Co doping which decreased from 6.0 µg mL^−1^ to 0.2 µg mL^−1^ (Δ~97%, 30-fold) at 40% of replacement of Fe^2+^ by Co^2+^. In addition, the nanotherapeutics based on Co40-MION demonstrated similar activity in killing GBM cells to that of chemotherapeutic drug standard DOX (0.2 µg mL^−1^) [1]. In this view, the in vitro results of the MTT bioassays using a 2D cell culture may be considered a breakthrough, indicating a prospective future clinical therapeutic application of Cox-MION nanozymes for brain cancer treatment to avoid the frequent side effects of DOX chemotherapy under systemic administration.

In the previous section (Section 3.2), the peroxidase-like activity of Cox-MION with the generation of highly oxidative hydroxyl radicals was demonstrated. One way to estimate the intracellular level of ROS is by using fluorogenic probes to measure the presence of these oxidative species directly. In this sense, aiming at measuring the accumulation of ROS generated in vitro inside GBM cells as a result of the biocatalytic activity of the nanozymes upon internalization, the relative amount of reactive species was found using 5-(and -6)-carboxy-2′,7′-dichlorodihydrofluorescein diacetate (DCF-DA).

This reagent was hydrolyzed to dichlorodihydrofluorescein (DCHF) after cell uptake and was converted to a green fluorescent emitter (2′,7′-dichlorofluorescein, DCF) in contact with the H_2_O_2_, hydroxyl, and peroxyl radicals [40].

**Figure 8:**
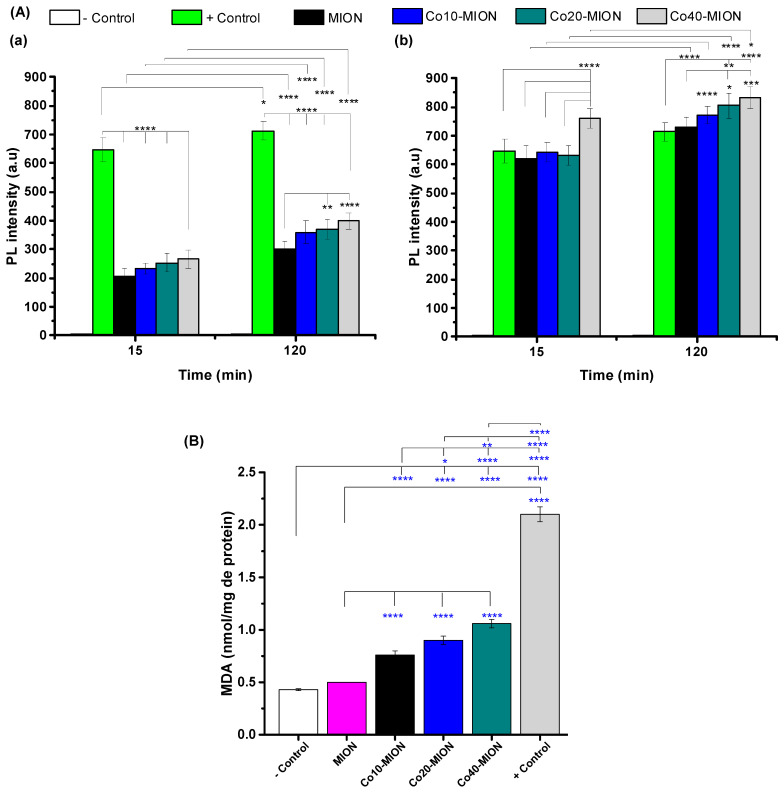
(**A**) Effect of cobalt content on ROS accumulation in U87 cells at 15 min and 2 h after induction by nanozymes at concentrations of (**a**) 0.6 µg mL^−1^ and (**b**) 6 µg mL^−1^ of Fe + Co compared to DCF + cells (“−”negative control) and TBHP (positive “+” control). (**B**) Level of lipid peroxidation in U87 cancer cells after 24 h of treatment with nanozymes at a concentration of 6 µg mL^−1^ (results are expressed in terms of MDA concentration as nanomol/mg of protein). Statistical analyses of ‘one-way’ ANOVA, Bonferroni, and multiple comparisons: **** = *p* < 0.0001, *** = *p* < 0.001, ** = *p* < 0.01, and * = *p* < 0.05 (*n* = 5 for ROS studies and *n* = 3 for MDA content).

The results for the PL intensity are presented in Figure 8A (15 and 120 min) and Figure S4 (15, 30, 60, and 120 min), and, as a general trend, a significant increase in intracellular ROS formation was observed compared to the negative control for both concentrations of the nanozymes tested ((a) 0.6 μg mL^−1^ and (b) 6 μg mL^−1^ of active Fe + Co). The values for the PL intensity for the nanozymes at the lower concentration (0.6 μg mL^−1^) were about three-fold smaller than that of the positive control (tert-Butyl hydroperoxide, TBHP) after 15 min of incubation of the U87 cells and, at a concentration of 6 μg mL^−1^, the fluorescence intensity was similar to or higher than the measured TBHP. This behavior indicated that the intracellular formation of ROS was clearly dependent on the nanozyme concentration, as the increase in the nanozyme concentration from 0.6 to 6 μg mL^−1^ resulted in a significant enhancement of the ROS formation. Additionally, the biocatalytic activity of Cox-MION nanozymes presented a similar trend to that observed for the catalytic behavior of the previous acellular results (i.e., without cells): an increase in the generation of ROS with the increase in Co content. This dependence of ROS formation on the concentration of the nanozyme and the Co-doping was in agreement with the cell viability results. This effect was expected because the presence of these species results in oxidative stress, which can induce cell death.

When analyzing the kinetics of intracellular ROS generation, an increase in the time of contact of the cells with Cox-MION nanoconjugates resulted in a higher PL intensity. Notably, the high DCF fluorescence measured after the first 15 min was assigned to hydrogen peroxide in the cells, which is far higher in cancer cells than in normal tissues [58]. As the specific substrate, it triggers the intracellular biocatalytic process with the presence of Fe(II), Co(II), and Fe(III) from iron oxide-based nanozymes mimicking peroxidase enzyme-like activity (POD). Considering the low PL value of the “−control” (DCF + U87 cells), the contribution of the intracellular H_2_O_2_ for fluorescence could be neglected in the absence of a nanocatalyst. In addition, after the rapid increase in the PL in the first 15 min, the reduction in the rate of increase in fluorescence may be attributed to the antioxidant mechanisms of the cell to counteract free radicals and neutralize oxidants. For example, for the Co40-MION nanozyme (at 6 μg mL^−1^), in the absence of the biocatalyst, the PL intensity was below 10 a.u. After 15 min of contact, a value of 760 a.u. was measured, corresponding to about 91% of the DCF signal observed after 120 min (832 a.u.).

Another alternative approach to evaluating oxidative stress relies on indirectly measuring the oxidative damage caused by ROS to the cell components, including lipid peroxidation (LPO) [40].

The LPO process damages the phospholipids of cell membranes by altering their physical properties, structure, and activity, which can induce a non-apoptotic cell death mechanism referred to as ferroptosis [66,67]. An assessment of the lipid damage was selected as ferroptosis involves the enhancement of ROS content associated with the presence of intracellular iron species and the depletion of the endogenous antioxidant glutathione (GSH). The LPO process mechanism was evaluated by malondialdehyde (MDA) protocols using thiobarbituric acid (TBA). The results of LPO in the presence of 6 µg mL^−1^ of nanozymes (Fe + Co related concentration), in the absence of cobalt doping (MION nanozyme), showed that the occurrence of significant lipid peroxidation was not observed as the MDA biomarker content was similar to that of the “−”control sample. On the contrary, the MDA content was significantly increased after incubation with Co-doped nanozymes, which was even enhanced at higher cobalt doping contents. These findings also demonstrated the generation of ROS promoted by the Co-MIOn nanozymes after internalization, corroborating the occurrence of ferroptosis-induced cell death [68,69].

In summary, based on the results obtained from the acellular analysis (the oxidation of TMB and spin-trapping EPR; see Section 3.2) combined with the 2D in vitro monolayer cell assays (staining of ROS with DCF and lipid peroxidation; this section), the cytotoxicity of the Cox-MION nanoparticles was mostly attributed to the generation of intracellular hydroxyl radicals (e.g., •OH). This effect confirmed the peroxidase-like biocatalytic activity of the iron-oxide-based nanozymes in the presence of endogenous hydrogen peroxide, which is present in higher levels in cancer cells. The excess of these species promotes an imbalance of ROS in the cells, resulting in cell death by the combined mechanisms of apoptosis and ferroptosis. The dependence of Co content was observed in all of the tests, and it is related to the higher redox potential of cobalt compared to iron, which favors the H_2_O_2_ decomposition mediated by the Cox-MION systems increasing the production of •OH-oxidizing radicals by doped nanozymes.

#### 3.3.2. 3D Cell Culture Models—Tumor Spheroids

##### Tumor Growth Evaluation

**Figure 9:**
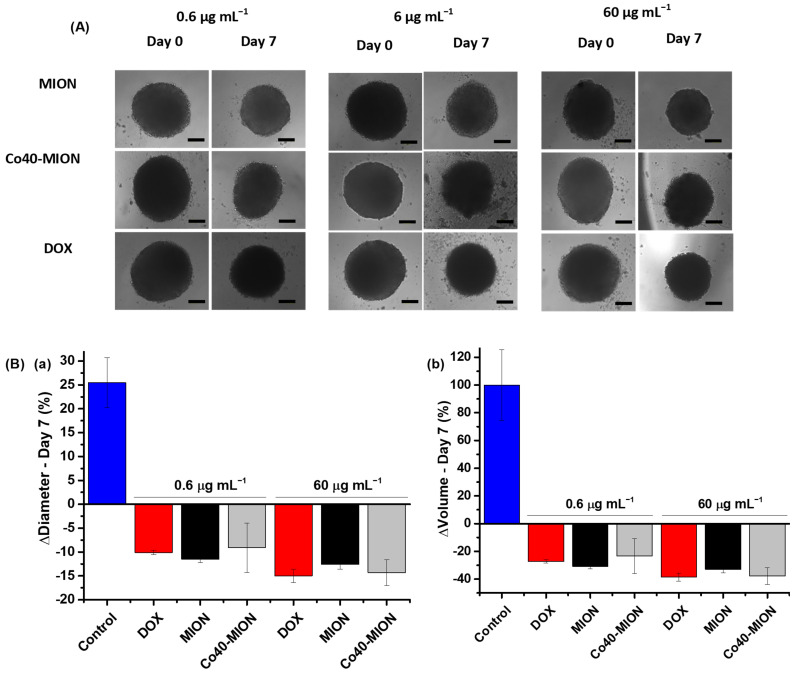
(**A**) Bright field images of the GBM spheroids before (Day 0) and after 7 days (Day 7) of treatment with different concentrations (0.6 and 60 μg mL^−1^) of MION, Co40-MION, and DOX (scale bar = 200 μm). (**B**) Changes in (**a**) diameter and (**b**) volume of tumor spheroids after 7 days of treatment (average ± standard error, SE).

Digital images were captured during the culture period to evaluate the size consistency and growth of tumor spheroids. After 10 days, the U87 tumor spheroid had distinct boundaries, a round and compact shape, and an average diameter of 711 μm (Figure 9A, Day 0). In the sequence, an intermittent dosing method with treatment on the first day and separated by three days of recovery before the administration of the second dose (as previously described in Figure 1) was tested.

Based on the previous results obtained by the 2D cell culture, the treatments were performed using MION (undoped sample, Co = 0%) and Co40-MION nanozymes (i.e., a lower EC-50 parameter and a higher generation of ROS and LOP damage in the monolayer cell culture), and DOX as a drug model at different doses (0.6, 6, and 60 μg mL^−1^). On the third day of the first treatment (identified as Day 3) and on the seventh day of the first treatment (identified as Day 7), bright field images were captured from the treated groups and control group (i.e., without treatment). The diameters and volumes of the 3D spheroids were quantified to investigate the activity of nanozymes on tumor growth.

As shown in the images presented in Figure 9A-Day 7, after 7 days of treatment, regardless of the concentration, all of the treatments with nanozymes (Co-doped or undoped MION) and DOX resulted in the dimensional reduction in the tumor spheroid and, in the absence of the therapy, the control group still grew. The treatments with 0.6 and 60 μg mL^−1^ of the nanozymes (MION and Co40-MION) resulted in spheroid diameters (Figure 9(Ba)) that were decreased by ~9% and ~14%, respectively, similar to DOX (0.6 μg mL^−1^, 11%, and 60 μg mL^−1^, 15%). On the contrary, the control sample’s average diameter increased by ~25%.

When dealing with volume changes of the tumors (Figure 9(Bb)), the spheroid control volume increased by 100% (doubled in size).

Remarkably, the spheroids treated with the novel nanozymes (average values of MION and Co-MION) were reduced by ~27% (0.6 μg mL^−1^) and ~36% (60 μg mL^−1^), indicating that the nanotherapeutics based on the nanozymes inhibited the tumor growth and considerably reduced their size by approximately one-third.

Although an undisputable trend was observed of a bigger reduction in tumor spheroid size at higher concentrations, it was not possible to state that it was statistically significant (‘one-way’ ANOVA and Bonferroni test) when considering the dependence of the reduction in diameter/volume with the dose in the treatment (0.6, 6, and 60 μg mL^−1^).

**Figure 10:**
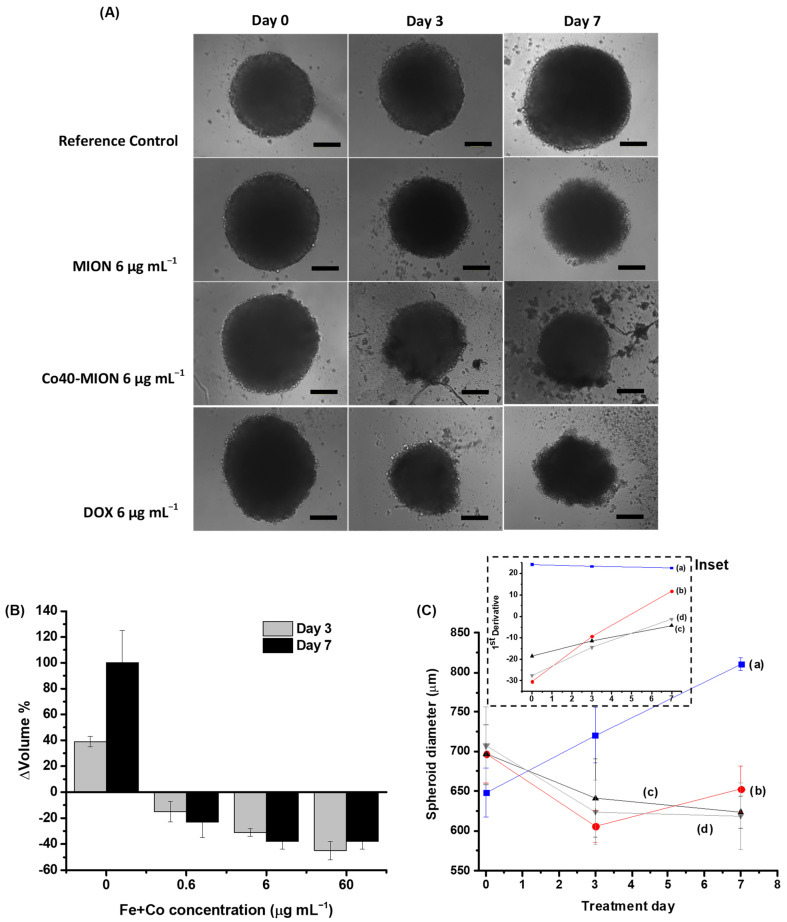
(**A**) Bright field images of tumor spheroids before and after 3 and 7 days of the first treatment dose (scale bar = 200 μm). (**B**) Volume reduction in spheroids treated with two doses of Co40-MION at different concentrations (average ± SE). (**C**) Evolution of spheroid diameter with time after treatment at 6 µg mL^−1^ ((a) Reference control, (b) DOX, (c) MION, and (d) Co40-MION, average ± SE, inset first derivative).

Likewise, similar tumor growth inhibitions were observed for both types of treatment, i.e., chemotherapy using a DOX anticancer drug and biocatalytic nanozyme-based therapy. To evaluate the effect of successive treatments on the size of GBM spheroids, bright field images were captured on Day 3 (before the second treatment) and Day 7. These images are displayed in Figure 10A (MION, Co40-MION, and DOX at 6 μg mL^−1^), and a quantitative analysis was performed based on the changes in their diameter measurements. When comparing the effect of retreatment, it could be observed that the shrinkage of the spheroid was expressive after 3 days of the first treatment (Day 3). Still, no significant additional size reduction was observed after 4 days of the second dose (Day 7). As shown in Figure 10B for Co40-MION, the 0.6 μg mL^−1^ and 6 μg mL^−1^ concentrations of the cobalt-doped nanozyme reduced the spheroid volume by 15% and 31%, respectively, after the first treatment. However, the same nanozyme concentration decreased the volume by 23% (0.6 μg mL^−1^) and 33% (6 μg mL^−1^) after the second dose. For 60 μg mL^−1^, an increase in the volume (Δvolume = 45% at 3 days and 38% at 7 days) was also detected. The first derivative of the spheroid diameter versus the time (inset Figure 10C) clearly showed that the cellular aggregates without treatment grew at a constant rate (~23 μm/day) during the 7 days. Conversely, the rate of decrease in size (i.e., kinetics) for the treated 3D systems slowed down, which is consistent with the characteristics of the tumor model, including nutrient diffusion, morphological features, and the formation of layers of dead cells. However, in 7 days (2 doses), important spheroid morphology/phenotype changes were observed. Some spheroids appeared disintegrated, with a more irregular spherical-like morphology, and had cells detached from the main tumor body due to the treatment. Such an effect could explain the absence of observed concentration-dependent responses [70].

##### Evaluation of Cell Viability of Tumor Spheroid Model

In order to evaluate the combined effect of the dimensional and morphological changes in the cell viability of the spheroids, additional tests were performed to improve the quantification of the nanotherapeutic strategy as an anticancer agent. The MTT test was selected, according to Ho et al. [71], as it could be potentially applied to high-throughput screening for spheroid cultures’ response to cytotoxic stimuli. Before the analyses on Day 3 and Day 7, the spheroid cultures were disaggregated into a single-cell suspension using enzymatic dissociation [43].

**Figure 11:**
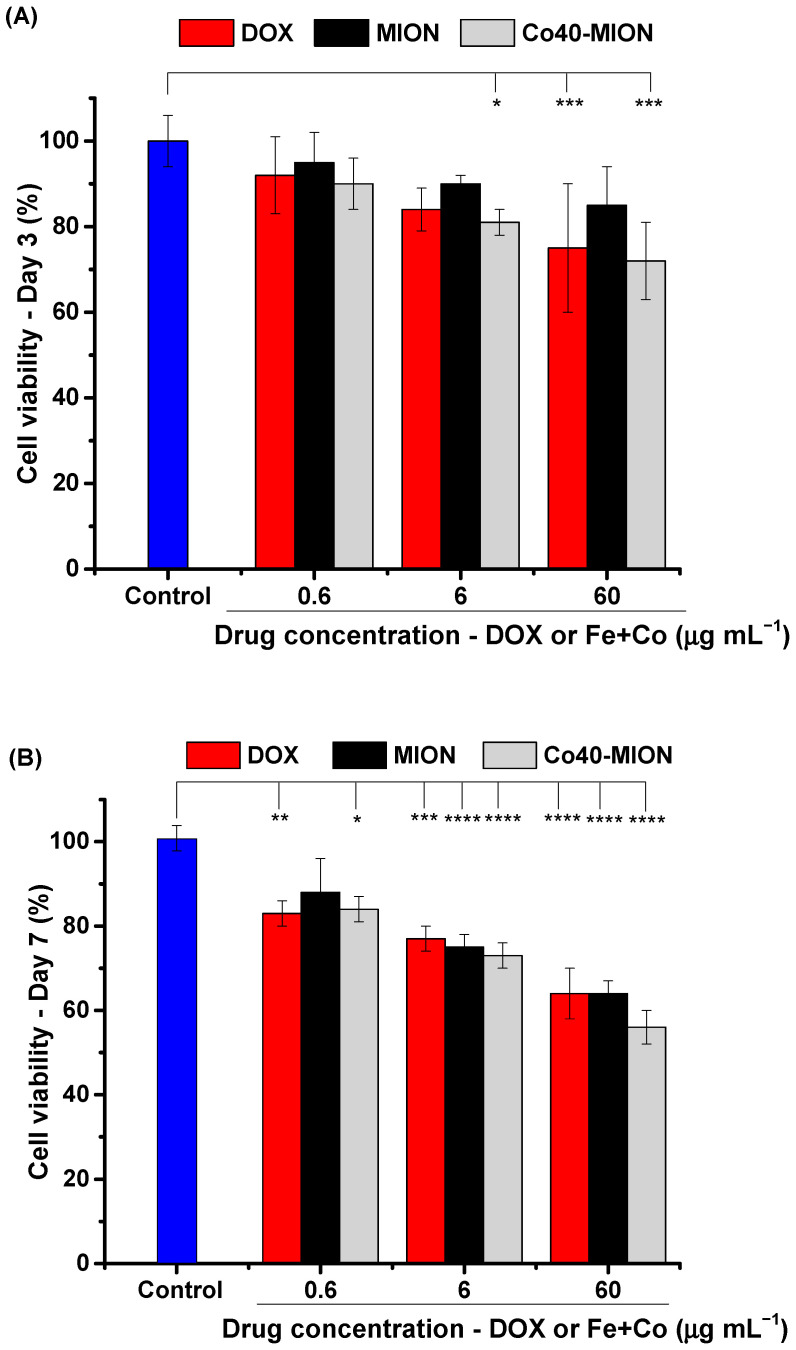
Cell viability (MTT assay) of glioblastoma spheroids treated for (**A**) 3 days and (**B**) 7 days with doxorubicin, MION, and Co40-MION nanozymes at different concentrations (average ± SD). Statistical analyses (related to control) of ‘one way’ ANOVA, Bonferroni, multiple comparisons: **** = *p* < 0.0001, *** = *p* < 0.001, ** = *p* < 0.01, and * = *p* < 0.05 (*n* = 3).

The cell viability results are presented in Figure 11A,B for tests after 3 days (effect of the first treatment) and 7 days (effect retreatment), respectively. After three days of treatment (Figure 11A), the number of cancer cells in the spheroids was significantly reduced for both Co40-MION and the standard drug. Additionally, the higher killing effect of cobalt doping verified in 2D cell cultures was also revealed in clustered cells at concentrations of 6 and 60 μg mL^−1^ (concentration and type of treatment effect).

After the retreatment (7 days, Figure 11B), the outcome was an increase in the antiproliferative properties of the nanozymes and DOX at all concentrations assayed with an enhancement in the statistical significance level at higher concentrations. The results meaningfully differed from those of the control, indicating the cell viability was dependent on the concentration of the anticancer agents, i.e., the nanozymes and doxorubicin.

Hence, the cell viability tests visibly pointed to the antiproliferative effects of the second dose and the dependence on concentration. In addition, this bioassay demonstrated important differences between the nanozymes and the drug as anticancer agents. In this sense, the cell viability analysis of the spheroids was crucial when evaluating novel therapeutic agents combined with the studies of the dimensional and morphological features of the tumor spheroids upon treatment. Despite being less sensitive, the size-based analysis of spheroids in anticancer drug studies reduces the use of biochemical assays and helps us identify effective treatments that promote shrinkage or prevent malignant tumor growth.

### 3.4. Effect of 2D and 3D Cell Cultures on Cell Viability Responses

**Figure 12:**
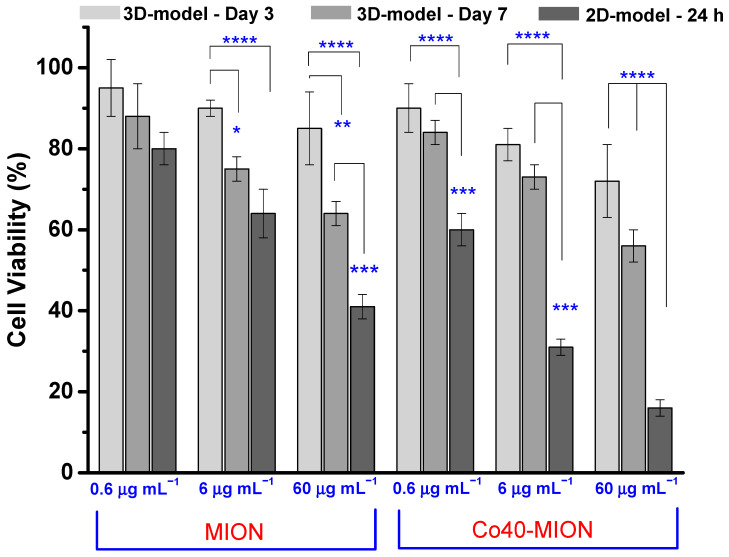
Cell viability of U87 cells grown in 2D monolayer and spheroid model after treatment with nanozymes (MION and Co40-MION) measured by the MTT assay (average ± SD). Statistical analyses of ‘one way’ ANOVA, Bonferroni, multiple comparisons: **** = *p* < 0.0001, *** = *p* < 0.001, ** = *p* < 0.01, and * = *p* < 0.05 (*n* = 3).

The comparison between the results of cell viability obtained from the 2D cell culture and the 3D model based on spheroids (Figure 12) indicated that the monolayer system was more susceptible to therapy activity, as usually reported in the literature [43,72]. On the other hand, the 3D spheroid GBM models were more resistant to the nanozyme treatment than their 2D monolayer counterparts. The 2D model, even with the lower time of treatment (24 h), caused relatively lower cell viability responses (i.e., higher cytotoxicity) at the same dose of the chemotherapeutic agent (compared to the 3D model after 3 days of spheroids) and even after the second dose (compared to the 3D model after 7 days of spheroids). Such a trend is usually associated with the fact that tumor cells in spheroids may spontaneously develop resistance to drugs, termed ‘‘multicellular resistance” (MCR). This characteristic has been associated with multicellular tissue architecture [71,73]. Nonetheless, the diffusion barrier to the drug penetration in the tumor spheroids should not be neglected compared to the monolayer culture due to high cell density, compaction, and heterogeneity, with the outer cells more exposed to treatments than the inner layers [72,74]. However, in the case under evaluation in this work, the effect of increasing Co-doping in the nanozymes in promoting the greater killing of GBM cells presented a similar trend for both models, indicating a matching response pattern. Additionally, it did not alter the dosage-dependent cytotoxicity observed for the nanozymes.

Based on the results, it may be affirmed that the 2D cell culture model for testing the nanozymes’ biocatalytic activity in glioblastoma cancer treatment was efficient in screening the most effective Co-doped iron oxide system. These findings are vital for the next step of our research in the spheroid model of GBM, currently considered the gold standard for glioblastoma in vitro studies [74]. Thus, although not considered the most appropriate model to predict the precise action of anticancer treatments coherently, the 2D monolayer system is easy and faster, low cost, and valuable for preliminary studies. In the sequence, tests using 3D tumor spheroids demonstrated the efficacy of nanozymes in eliminating brain cancer cells, similar to the DOX model chemotherapeutic drug. The 3D spheroid model incorporates the important features of solid tumor masses, such as the TME, cell–cell interactions, expression of antigens, nutrients and oxygen gradients, and distribution of proliferating/quiescent/necrotic cells within the spheroid, among others [70,71,72,73,74].

It is still expected that this 3D model based on tumor spheroids could be further improved and more reliable. For instance, co-cultures may be used that more closely mimic the physiological context of the malignant tumor, or cell clusters from patient-derived cell cultures (PDCs) obtained from cancer surgical specimens could be used, which could preserve genomic alterations in epidermal growth factor receptors [74,75]. This development will certainly lead to models that more successfully resemble in vivo conditions, increasing the chance for reliable predictive preclinical behavior instead of the current animal models that present numerous limitations, such as ethical concerns, higher costs and time consumption, and inability to represent complex organs and tissues [76,77,78,79]. Moreover, it should be highlighted that, although very promising, these findings associated with nanozymes cannot be directly applied to clinical use in brain cancer therapy. Several additional aspects must be taken into account through translational medicine strategy, including biochemical stability for systemic administration, biodistribution to overcome the blood–brain barrier (BBB), and possible off-target effects before reaching the tumor site [80]. In addition, like most natural enzymes, the biocatalytic activity of nanozymes can be affected by numerous complex and dynamic biological interactions at the organ, tissue, and cellular levels, including blood and components, which can improve, deteriorate, temporarily inhibit, or permanently inhibit their enzyme-like characteristics [81,82,83,84,85,86,87].

Nonetheless, this study relying on 2D and 3D models using glioma and healthy cell lines demonstrated promising results to support conditions of future safe clinical use while additionally favoring an improvement in the effectiveness of cancer therapy by modulating intracellular ROS generation targeting tumor sites.

## 4. Conclusions

In summary, in this study, as hypothesized, we presented the rational design and synthesis of novel nanostructures composed of cobalt-doped iron oxide nanoparticles, with increasing cobalt contents from 0% to 40%, which were chemically stabilized by carboxymethylcellulose acting as the capping ligand (Cox-MION). These nanoconjugates were produced using a strictly green colloidal aqueous process under mild conditions for creating non-toxic bioengineered peroxidase-like (POD) nanozymes for the biocatalytic killing of GBM cancer cells. These nanozymes (Cox-MION) were extensively characterized, demonstrating a crystalline inorganic core of nanosized magnetite with a uniform spherical morphological aspect (2R = 6–7 nm), capped by the CMC biopolymer shell. They exhibited an overall hydrodynamic diameter (H_D_) of 41–52 nm and a negative zeta potential surface charge of ZP = −50 mV, displaying supramolecular water-dispersible colloidal nanostructures. Moreover, the nanozymes confirmed the cytotoxicity against U87 brain cancer cells evaluated using 2D cultures in vitro (i.e., the MTT bioassay), which was concentration-dependent and boosted by increasing the cobalt doping content in the nanosystems. The results demonstrated that the lethality of U87 cancer cells was biocatalytically activated by the nanozymes displaying POD-like behavior, producing highly toxic reactive oxygen species (ROS) by generating hydroxyl radicals (·OH). The results demonstrated that the nanozymes induced the ferroptosis (i.e., lipid peroxidation) pathways by intracellular biocatalytic enzyme-like activity. More importantly, based on the 3D spheroids model, these nanozymes hampered the tumor growth and, remarkably, decreased the malignant tumor volume after nanotherapeutic treatment by approximately 40%. Similar to the tendency often observed in tumor microenvironments, the 3D GBM spheroidal models showed a decreasing rate of anticancer activity with the incubation time with the novel nanotherapeutics. Additionally, the results demonstrated that the 2D in vitro model overestimated the relative efficiency for killing cancer cells (i.e., nanozymes and the DOX drug) compared to the 3D spheroid models. These findings are outstanding as they validated our hypothesis that the 3D spheroid model resembles the TME of “real” brain cancer tumors in patients more precisely than 2D cell cultures, which may lead to inaccurate dosage-killing evaluations. In this view, it can be anticipated that biological 3D tumor spheroid models offer a prospective alternative as a reliable intermediate system between traditional 2D cell cultures and complex in vivo animal models for evaluating anticancer agents more precisely. These nanotherapeutics can offer a flexible nanoplatform to create an arsenal of innovative nanomedicines to fight cancer and reduce the harsh side effects of chemotherapy.

## Figures and Tables

**Table 1:** Co content in Cox-MION (Co_x_Fe_3−x_O_4_) nanozymes.

Sample	Co Content (%mol)	
	Related to Fe^2+^	
	x	x%
MION	0.0	0%
Co10-MION	0.1	10%
Co20-MION	0.2	20%
Co40-MION	0.4	40%

## Data Availability

All relevant data are available in the manuscript or the supporting information.

## Supplementary Materials

The following supporting information can be downloaded at: https://www.mdpi.com/article/10.3390/pharmaceutics15061702/s1, Table S1—Crystallite size based on XRD data (Scherrer’s Equation); Figure S1—Evolution of atomic concentration of (a) Fe^2+^ and (b) Co^2+^ with increasing cobalt doping content; Figure S2—Diameter of nanozyme inorganic core as a function of Co content; Figure S3—(A) Hydrodynamic diameter (columns) and Zeta potential (symbols/line) of nanozymes. (B) Zeta potential values of CMC as a function of pH; and Figure S4—Effect of cobalt content on ROS accumulation in GBM cells at 15 min, 30 min, 60 min, and 120 min after contact with nanozymes at concentrations of (A) 0.6 µg mL^−1^ and (B) 6 µg mL^−1^ of Fe + Co compared to DCF + cells (“-”negative control) and TBHP (positive “+” control).
