# Supplementary material for: Nanozymes with Peroxidase-like Activity for Ferroptosis-Driven Biocatalytic Nanotherapeutics of Glioblastoma Cancer: 2D and 3D Spheroids Models
Source: Pharmaceutics. 2023 Jun 10;15(6):1702. doi: 10.3390/pharmaceutics15061702 (PMC10301181; doi:10.3390/pharmaceutics15061702)
Supplement: Supplementary file 1 [file pharmaceutics-15-01702-s001.zip › pharmaceutics-2349886-supplementary.pdf]

pharmaceutics

Tables

Table S1. Crystallite size based on XRD data (Scherrer's Equation) (average ± standard deviation).

Sample	Crystallite size (nm)	
	Plane (311)	Plane (220)
MION	6.4 ± 0.6	6.4 ± 0.3
Co10-MION	6.6 ± 0.9	6.5 ± 0.5
Co20-MION	6.5 ± 1.0	7.3 ± 0.2
Co40-MION	6.1 ± 0.1	5.4 ± 0.4

Figures

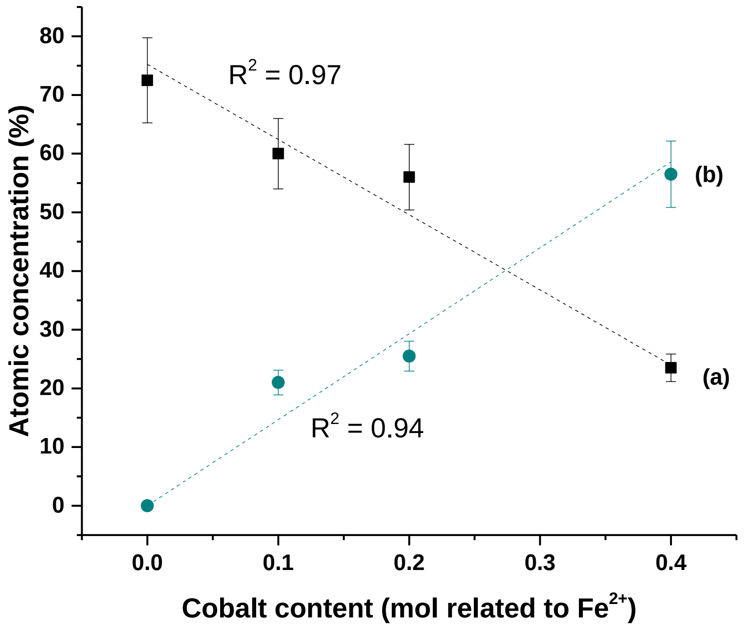

Figure S1. Evolution of atomic concentration of (a) Fe^{2+} and (b) Co^{2+} with increasing cobalt doping content.

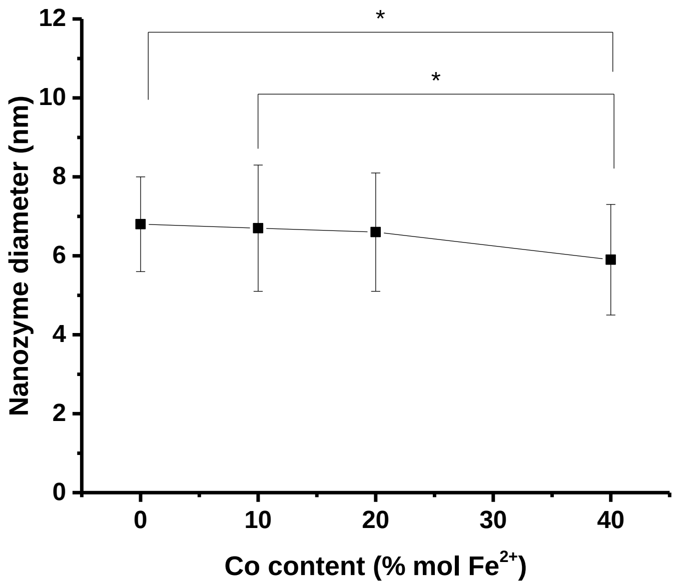

Figure S2. Diameter of nanozyme inorganic core as a function of Co content.

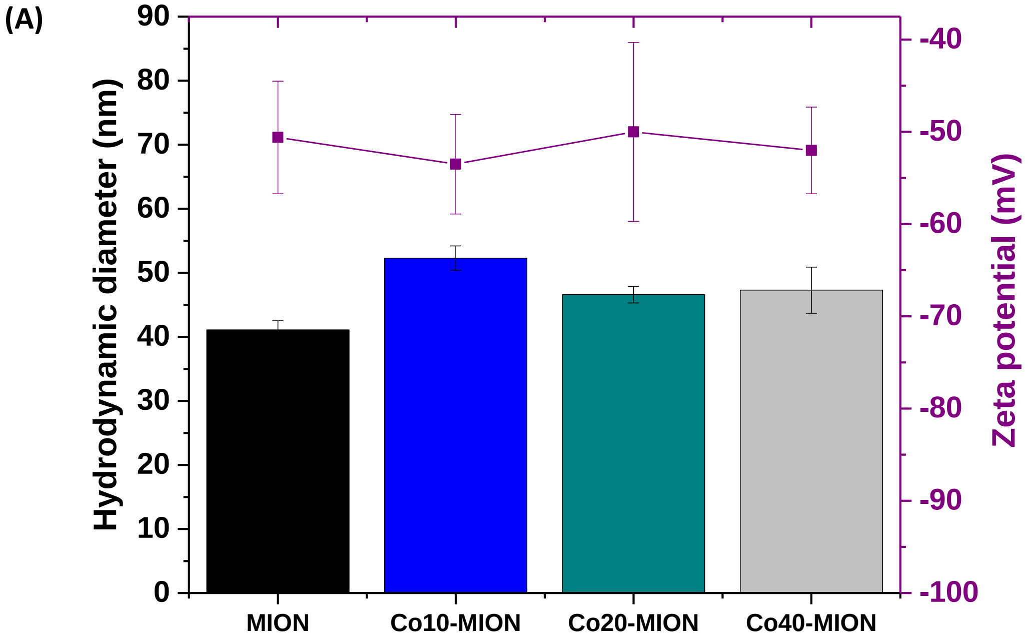

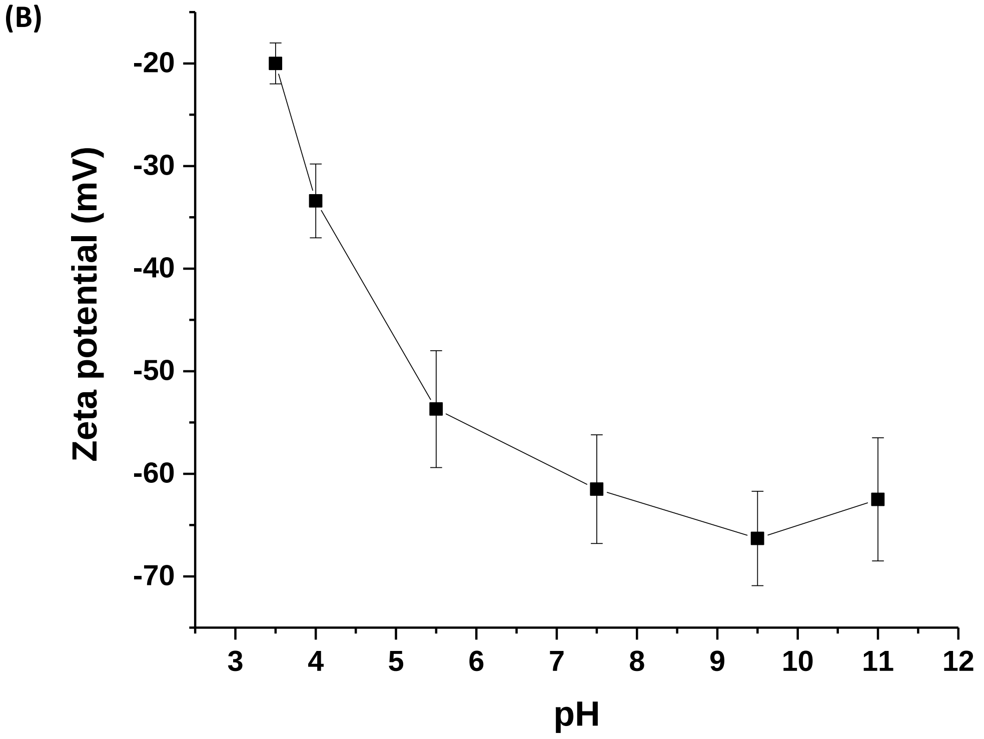

Figure S3. (A) Hydrodynamic diameter (columns) and Zeta potential (symbols/line) of nanozymes (average ± standard deviation, n≥3). (B) Zeta potential values of CMC as a function of pH (average ± standard deviation, n≥3).

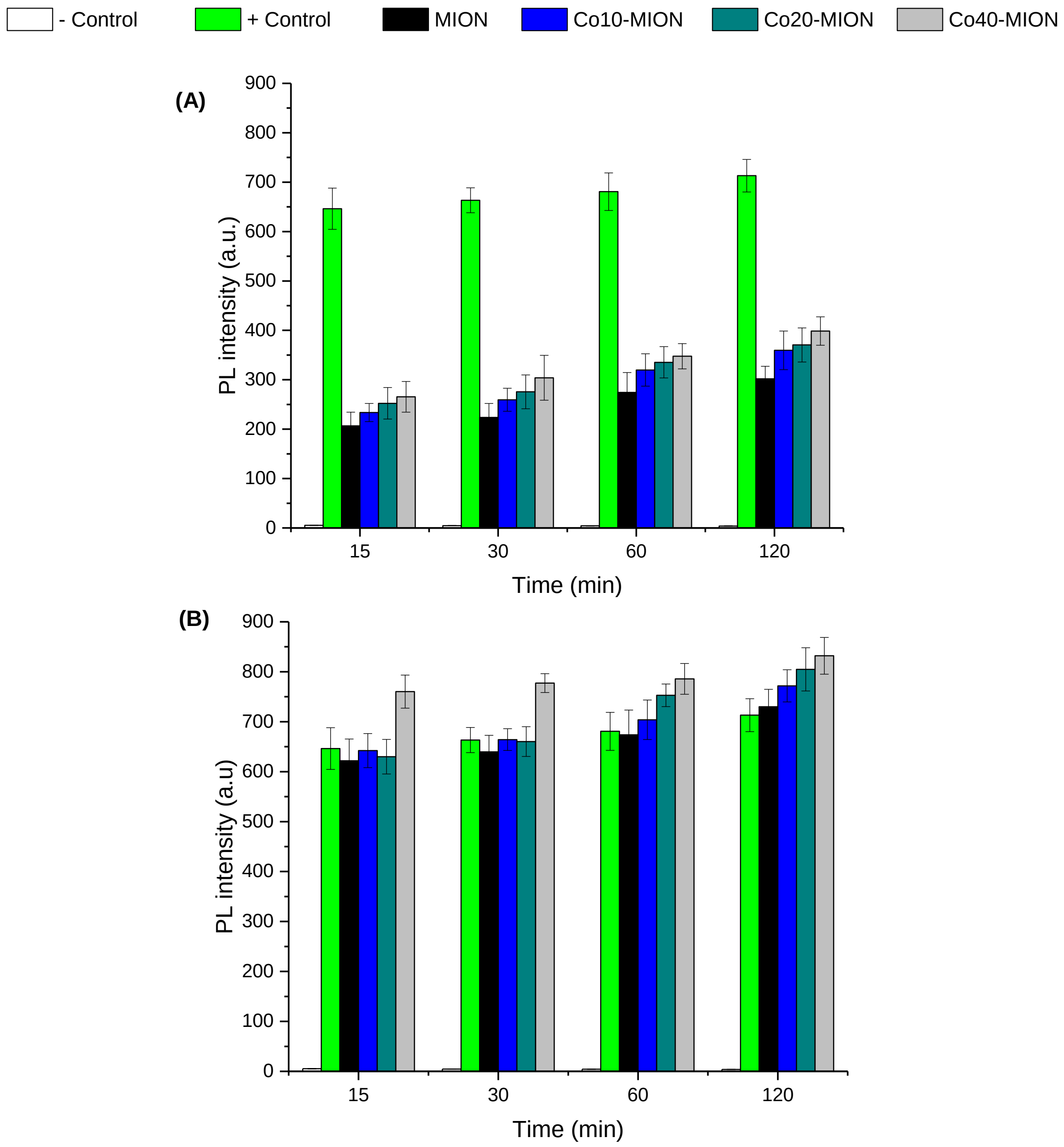

Figure S4. Effect of cobalt content on ROS accumulation in GBM cells at 15 min, 30 min, 60 min, and 120 min after contact with nanozymes at concentrations of (A) 0.6 μg mL^{-1} and (B) 6 μg mL^{-1} of Fe+Co compared to DCF+cells ("−"negative control) and TBHP (positive "+" control). (average ± standard deviation, n=5).
